# Radiotherapy Enhances the Oncolytic Efficacy of the Novel Oncolytic Herpesvirus VG161 and Amplifies Its Antitumor Immunity in Breast Cancer

**DOI:** 10.1002/advs.202523356

**Published:** 2026-06-29

**Authors:** Lijuan Lyu, Ming Yi, Ji Chen, Zeda Zhao, Ruocen Liao, Yingnan Wang, Shengtao Hu, Jun Ding, Ronghua Zhao, William Jia, Yinan Shen, Yongchang Wei, Kongming Wu, Zhijun Dai

**Affiliations:** ^1^ Department of Breast Surgery College of Medicine The First Affiliated Hospital Zhejiang University Hangzhou China; ^2^ Department of General Surgery The Fourth Affiliated Hospital of Soochow University (Suzhou Dushu Lake Hospital) Suzhou China; ^3^ Department of Radiation and Medical Oncology Hubei Key Laboratory of Tumor Biological Behaviors Hubei Cancer Clinical Study Center Zhongnan Hospital of Wuhan University Wuhan China; ^4^ Zhejiang Provincial Key Laboratory of Pancreatic Disease Hangzhou China; ^5^ Shanghai Virogin Biotech Co., Ltd. Shanghai China; ^6^ Department of Hepatobiliary and Pancreatic Surgery College of Medicine The First Affiliated Hospital Zhejiang University Hangzhou China; ^7^ Cancer Center Shanxi Bethune Hospital Shanxi Academy of Medical Science Tongji Shanxi Hospital Third Hospital of Shanxi Medical University Taiyuan China; ^8^ Cancer Center Tongji Hospital of Tongji Medical College Huazhong University of Science and Technology Wuhan China

**Keywords:** antitumor immunity, breast cancer, oncolytic virus replication, radiotherapy, VG161

## Abstract

VG161 is an oncolytic HSV‐1 with an ICP34.5 deletion and armed with multiple immunomodulatory factors. The absence of ICP34.5 restricts viral replication in neurons while conferring tumor specificity. However, this modification attenuates viral replication in tumor cells compared to wild‐type HSV‐1. Radiotherapy (RT) has been reported to promote viral replication and exert an immune‐priming effect. Based on this, we hypothesize that RT could potentiate both the VG161 replication and its antitumor efficacy in breast cancer (BC). Our findings suggest that the optimal regimen for combining VG161 with RT involves administering 5 Gy irradiation 6 h after VG161 infection, which ensures that RT maximally promotes VG161 replication in BC. This enhancement in VG161 replication is mediated by the upregulation of GADD34 and HVEM induced by RT. Moreover, RT augments the expression of immunostimulatory transgenes carried by VG161 and immunogenic cell death of BC cells. In vivo, VG161 combined with RT demonstrates superior antitumor efficacy compared with either monotherapy in BC. Mechanistic investigations reveal that this combination therapy increases the abundances of tumor‐infiltrating lymphocytes and elicits potent systemic antitumor immunity that inhibits local tumors and regresses abscopal metastases. The observed synergistic effect of VG161 and RT encourages further clinical translation.

## Graphical abstract

RT promotes VG161 replication in BC and its immunostimulatory transgenes expression, which is mediated by the upregulation of GADD34 and HVEM caused by RT. The combination therapy with VG161 and RT increases the abundances of tumor‐infiltrating lymphocytes and elicits potent systemic antitumor immunity, thereby effectively inhibiting local tumors and amplifying abscopal effect against preestablished, spontaneous lung metastases.

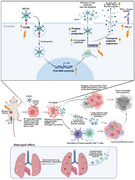

## 1 Introduction

Breast cancer (BC) remains a leading cause of cancer‐related mortality in women, reflecting profound disease heterogeneity, metastasis, and therapeutic resistance [1]. Oncolytic viruses (OVs) is an emerging therapeutic approach for BC that selectively replicates within and destroys tumor cells while sparing normal cells from damage [2]. Beyond direct oncolysis, OVs can initiate anti‐tumor immunity by inducing immunogenic cell death (ICD), leading to the release of tumor‐associated antigens and damage‐associated molecular patterns (DAMPs), and remodeling of the immune “cold” tumor microenvironment (TME) by expressing immune‐stimulating transgenes, making them a promising strategy to evoke antitumor immunity [3]. A diverse range of OVs has shown efficacy in preclinical studies [4, 5]. Only four OVs have been approved for use in humans as of 2025, half of which are engineered herpesviruses: talimogene laherparepvec (T‐VEC; IMLYGIC) is a modified herpes simplex virus type 1(HSV‐1) approved in the United States and Europe in 2015 for treating melanoma [6]; and DELYTACT (teserpaturev/G47D) is an attenuated HSV‐1 approved in Japan in 2021 for the treatment of primary brain cancer [7]. They are partially attenuated by deleting ICP34.5.

VG161 is a partially attenuated oncolytic HSV‐1 carrying a broad suite of immunomodulatory transgenes, including the secretable programmed cell death 1 ligand 1 (PD‐L1) blocking peptide controlled by the human EF‐1α promoter and the human IL‐12, IL‐15, and IL‐15 receptor alpha subunit isoform 1 (IL‐15RA) driven by the CMV promoter [8, 9]. We have previously evaluated VG161 both in vitro and in vivo, demonstrating a synergistic immunostimulatory interaction between the four payloads, resulting in robust antitumor efficacy in both human tumor xenografts and syngeneic mouse tumor models [10, 11]. VG161 has completed phase I clinical trials in Australia and China, demonstrating excellent safety and promising efficacy in patients with various solid tumors [12, 13]. Similar to IMLYGIC and G47D, VG161 lacks the major neurovirulence factor ICP34.5. The deletion of the HSV‐1 gene encoding ICP34.5 is a common feature of oncolytic HSVs designed for human use. ICP34.5 deletion prevents the virus from replicating in neurons while simultaneously imparting a measure of tumor specificity [14, 15]. However, this safety feature comes at a cost, resulting in approximately a 10‐fold reduction in virus replication for the ICP34.5‐deleted virus compared with the wild type. Therefore, it is necessary to identify a suitable combination therapy to improve the replication efficiency of ICP34.5‐deleted VG161 in tumor cells. Recent studies have suggested a synergistic antitumor effect of HSV‐1 when combined with radiotherapy (RT) [16, 17].

First used to treat cancer over a century ago, RT has become the mainstay of first‐line treatment for numerous solid tumors. In addition to mediating DNA damage, RT exerts profound immunostimulatory effects on the TME by triggering ICD and promoting the production of pro‐inflammatory cytokines and chemokines [18, 19]. Interestingly, RT has been reported to enhance the viral replication and antitumor efficacy of ICP34.5‐deleted HSV‐1 in preclinical tumor models and clinical trials [17, 20, 21, 22, 23, 24, 25, 26]. A preclinical study demonstrated that a single radiation dose of 5 Gy given 6–9 h after HSV‐1 injection resulted in maximal viral gene expression and infectious viral production and maximized synergistic effects in tumor xenografts [24]. Mechanistic studies have elucidated multiple pathways through which RT enhances viral replication. The HSV‐1 immediate‐early protein ICP0 inhibits DNA damage repair following the irradiation of glioma cells while hijacking repair signals to promote viral replication [27, 28]. RT also induces the upregulation of cellular genes that trans‐complement the functions of deleted viral genes. RT‐enhanced expression of cellular ribonucleotide reductase assists the replication of viruses lacking viral ribonucleotide reductase [29]. Another example of trans‐complementation involves HSV‐1 viruses deleted of ICP34.5. The mammalian homologue of ICP34.5 is the growth arrest and DNA damage 34 (GADD34) protein, which can functionally substitute for ICP34.5 [30]. GADD34 is upregulated following RT and has been shown to increase the replication of HSV‐1 that deletes ICP34.5 [31, 32, 33]. A third mechanism by which RT can enhance viral replication is through the increased expression of viral proteins via the upregulation of viral promoters. It has been shown that RT enhances transcription of late HSV‐1 viral promoters by activating the p38 mitogen‐activated protein kinase pathway [34]. Finally, radiation may synergize with oncolytic HSV‐1 by priming antitumor innate and adaptive immune responses.

In a previous study, we demonstrated that VG161 significantly repressed BC growth and elicited a robust antitumor immune response when combined with paclitaxel treatment in mouse BC model [35]. In this study, we hypothesize that the combination of RT with VG161 may have a potentiating effect on viral replication and antitumor immunity and attempt to test this hypothesis in preclinical studies, thus extending the future clinical applications of VG161 in BC therapeutics.

## 2 Results

### 2.1 Optimal Irradiation Dose and Timing for Promoting VG161 Replication in BC

**FIGURE 1:**
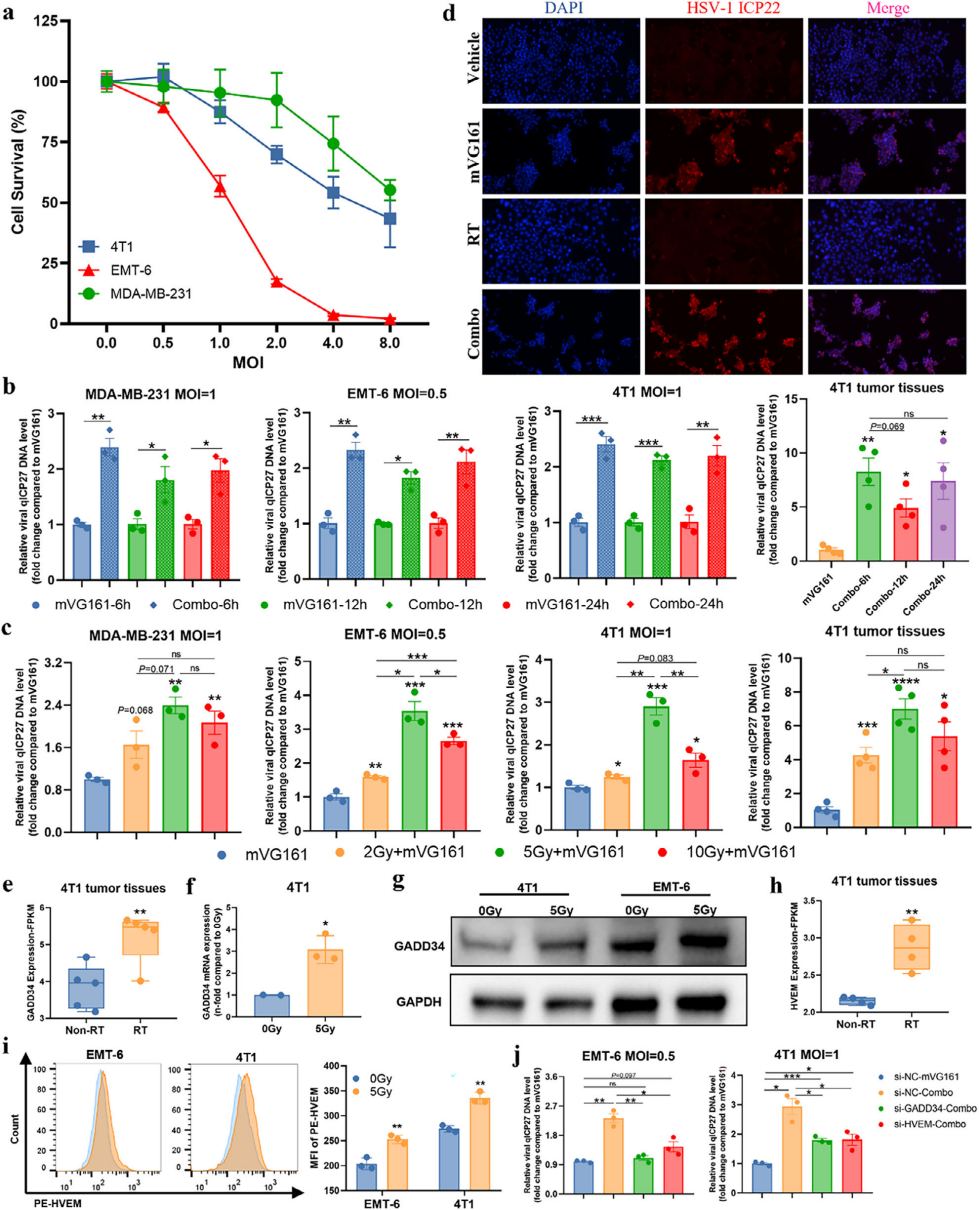
Delivery of a moderate dose of 5 Gy irradiation 6 h after VG161 infection promotes VG161 viral entry into BC cells and its intracellular replication by inducing HVEM and GADD34. (a) The cytotoxic effect of VG161/mVG161 was evaluated in MDA‐MB‐231, EMT‐6, and 4T1 cells at 48 h post infection and MOI of 8, 4, 2, 1, 0.5 (n = 4). (b) Tumor cells and 4T1 tumor tissues were irradiated with 5 Gy at 6, 12, and 24 h after infection with VG161/mVG161, respectively; 48 h later, genomic DNA was isolated and subjected to qPCR to quantify the relative level of viral qICP27 DNA (n = 3 or 4). (c) Tumor cells and 4T1 tumor tissues were respectively irradiated with 2, 5, and 10 Gy at 6 h after infection with VG161/mVG161; 48 h later, genomic DNA was isolated and subjected to qPCR to quantify the relative level of viral qICP27 DNA (n = 3 or 4). (d) The HSV‐1 ICP22 expression in 4T1 cells treated with 5 Gy or mVG161 only, or 5 Gy was given at 6 h following mVG161 infection, was detected by immunofluorescence staining at 24 h after different treatments. (e,h) The expressions of GADD34 and HVEM 2 days after irradiation (5 Gy * 2) or without irradiation in the 4T1 tumors were detected by RNA‐seq (n = 5 or 4). (f) qRT‐PCR was used to detect GADD34 expression in 4T1 cells at 5 h post‐irradiation (n = 2 or 3). (g) Western blotting assay to measure GADD34 expression in 4T1 cells and EMT‐6 cells at 4 h after irradiation or non‐irradiation. (i) Representative images and quantification of the HVEM expression on the surface of EMT‐6 and 4T1 cells were detected by flow cytometry at 24 h post‐irradiation or non‐irradiation (n = 3). (j) Tumor cells transfected with siRNA 24 h prior were infected with mVG161 and exposed to 5 Gy irradiation at 6 h post‐infection. 24 h after irradiation, genomic DNA was extracted and subjected to qPCR to quantify the relative level of viral ICP27 DNA (n = 3). All data are mean±SEM. The *p* values were calculated by Student's *t*‐test. Significance is indicated as: ^*^ *p* < 0.05, ^**^ *p* < 0.01, ^***^ *p* < 0.001, and ^****^ *p* < 0.0001. ns: not significant.

The oncolytic capability of VG161 was evaluated in human MDA‐MB‐231 and mouse EMT‐6 and 4T1 BC cell lines. The cells were infected with VG161/mVG161 for 48 h at a multiplicity of infection (MOI) ranging from 0.5 to 8, and cell viability was quantified using the CCK‐8 assay. VG161 exhibited robust cell‐killing ability in EMT‐6 cells, whereas its oncolysis was considerably diminished in 4T1 and MDA‐MB‐231 cells (Figure 1a). This finding indicates that different cell lines may exhibit varying degrees of sensitivity to VG161. Viral replication requires the temporal expression of viral genes, and RT has been previously demonstrated to enhance late viral promoter genes [24]. We hypothesized that there is an optimal time to deliver RT during the replicative cycle of oncolytic HSV‐1 to maximize viral replication. MDA‐MB‐231, EMT‐6, and 4T1 BC cells were irradiated with 5 Gy at 6, 12, and 24 h, respectively, after infection with VG161/mVG161, and the relative levels of viral qICP27 DNA were determined by qPCR. The results showed that in BC cells irradiated with 5 Gy at 6, 12, and 24 h after infection with VG161/mVG161, the relative viral qICP27 DNA levels were increased compared to those in cells infected with VG161 alone at the corresponding time points (Figure 1b). However, the increase in the relative viral qICP27 DNA level was greater in cells irradiated 6 h after VG161/mVG161 infection than in those irradiated 12 or 24 h after infection (Figure 1b). A similar finding was observed in the 4T1 tumor tissues (Figure 1b). We then determined whether an RT dose response to enhance VG161/mVG161 DNA replication existed. BC cells received low‐dose (2 Gy), medium‐dose (5 Gy), and high‐dose (10 Gy) irradiation 6 h after infection with VG161/mVG161. The results indicated that, compared to infection with VG161/mVG161 alone, all three irradiation doses increased the relative levels of viral qICP27 DNA in the BC cells. Medium‐dose (5 Gy) irradiation specifically resulted in a greater increase in the relative level of viral qICP27 DNA in the BC cells than low‐ and high‐dose irradiations (Figure 1c). Consistent results were obtained for 4T1 tumor tissues (Figure 1c). These findings suggest that the optimal regimen for combining oncolytic VG161 with RT involves administering 5 Gy irradiation 6 h after VG161 infection, as this ensures that RT maximally promotes VG161 replication in BC cells and tissues. Subsequent experiments were conducted using this optimal combination regimen. Furthermore, we employed immunofluorescence (IF) staining to verify whether the optimal combination regimen described above enhanced the expression of the VG161 viral protein. As depicted in Figure 1d, 4T1 cells treated with 5 Gy of irradiation 6 h after mVG161 infection showed upregulation of HSV‐1 ICP22 protein expression compared with mVG161 monotherapy (Figure 1d).

### 2.2 RT Enhances the Efficiency of VG161 Infection and Promotes Its Intracellular Replication by Inducing HVEM and GADD34

Subsequently, we investigated the mechanism by which radiotherapy promotes VG161 replication in BC cells. As previously reported, the mammalian homologue of the ICP34.5 gene product is the GADD34 protein, which can functionally substitute for the ICP34.5 protein and is upregulated during DNA damage. We postulated that upregulation of GADD34 caused by RT mediates the synergistic antitumor activity of RT and ICP34.5‐deleted oncolytic herpesvirus VG161. Analysis of RNA sequencing (RNA‐seq) data from irradiated and non‐irradiated 4T1 tumor tissues indicated that GADD34 expression was upregulated in irradiated tissues (Figure 1e). Subsequent qRT‐PCR and western blot assays confirmed that RT increased GADD34 expression in both 4T1 and EMT‐6 cells (Figure 1f,g). Analysis of RNA‐seq data from irradiated and non‐irradiated 4T1 tumor tissues also revealed that herpesvirus entry mediator (HVEM) was upregulated in irradiated tumor tissues (Figure 1h). HVEM is expressed on the surface of tumor cells and mediates herpesvirus entry into these cells. The increased expression of HVEM on the surfaces of 4T1 and EMT‐6 cells following RT was verified using flow cytometry (Figure 1i). Upregulation of HVEM on the surface of tumor cells can increase VG161 the efficiency of VG161 infection. To determine whether the RT‐induced VG161 increase in BC is mediated by its upregulation of GADD34 and HVEM, EMT‐6 and 4T1 cells were transfected with siRNA targeting these genes and were then treated with either combination therapy or mVG161 monotherapy. In cells transfected with the negative control siRNA (si‐NC), the relative levels of viral qICP27 DNA were significantly higher after combination therapy than after treatment with mVG161 alone (Figure 1j). Tumor cells treated with the combination therapy following GADD34 or HVEM knockdown exhibited significantly lower viral qICP27 DNA levels than those treated with the combination therapy following si‐NC transfection (Figure 1j). However, these levels were higher than those in tumor cells treated with mVG161 alone after si‐NC transfection (Figure 1j). These findings suggest that the increase in VG161 induced by RT in BC is partially dependent on the upregulation of GADD34 and HVEM caused by RT.

### 2.3 RT Enhances the Expression of the Immunomodulatory Transgenes Carried by VG161, Thereby Effectively Activating T Cells In Vitro

**FIGURE 2:**
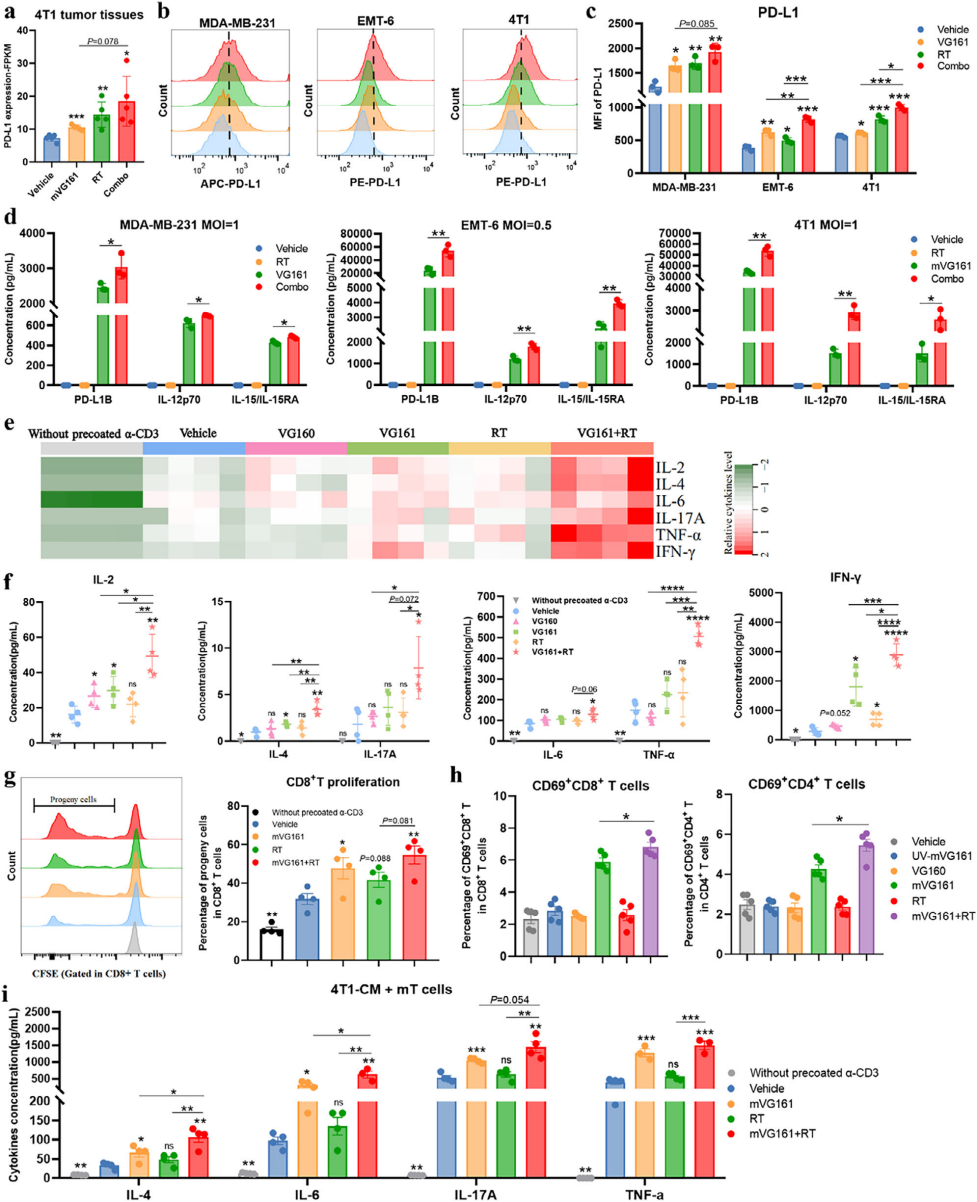
Irradiation enhances the expression of the VG161 transgenic payloads, which effectively activate T cells in vitro. (a) The PD‐L1 expression level in 4T1 tumor tissues 2 days after various treatments was detected by RNA‐seq (n = 5). (b,c) Representative images and quantification of the PD‐L1 expression on the surface of MDA‐MB‐231, EMT‐6, and 4T1 cells 24 h after different treatments were detected by flow cytometry, respectively (n = 3). (d) MDA‐MB‐231, EMT‐6, and 4T1 cells were treated with either monotherapy or combination therapy. After 48 h, the transgene expression products in the cell supernatants (PD‐L1 peptide blocker (PD‐L1B), IL‐12, and IL‐15/IL‐15RA) were detected using an ELISA or CBA (n = 3). (e,f) The effect of immunomodulatory payloads of VG161 on the cytokine secretion during T cell activation. Human T cells isolated from PBMCs were cocultured with supernatants of MDA‐MB‐231 cells treated with VG160, VG161, RT, or VG161 plus RT. After 3 days, the cellular supernatants were harvested to detect cytokine levels. The cytokine levels were visualized by a heatmap (n = 4). (g,i) The effect of immunomodulatory payloads of mVG161 on mouse T cell proliferation and activation. Mouse T cells isolated from splenocytes of BALB/c mice were cocultured with supernatants of 4T1 cells treated with either monotherapy or combination therapy. After culturing for 4 days, the percentage of progeny CD8^+^ T cells (g) and the cytokine levels in the supernatant (i) were measured (n = 4). (h) The effect of immunomodulatory payloads of mVG161 on mouse T cell activation. Mouse splenocytes from BALB/c mice were cocultured with supernatants of 4T1 cells treated with either monotherapy or combination therapy. After culturing for 2 days, the percentage of CD69^+^ CD8^+^ T cells and CD69^+^ CD4^+^ T cells were detected by flow cytometry (n = 5). All data are depicted as mean±SEM. Statistical analyses were conducted using Student's *t*‐test. Statistical significance compared to vehicle is indicated as follows: ^*^ *p* < 0.05, ^**^ *p* < 0.01, ^***^ *p* < 0.001, and ^****^ *p* < 0.0001. ns: not significant.

Previous studies have shown that RT can increase the expression of PD‐L1 in tumors, enabling cancer cells to evade immunotherapy [36]. We investigated the effect of mVG161 combined with RT on PD‐L1 expression in BC cells. Analysis of RNA‐seq data from 4T1 tumor tissues treated with either combination therapy or monotherapy showed that PD‐L1 was markedly upregulated in 4T1 tumor tissues treated with mVG161 or RT alone and further increased following the combination of mVG161 with RT compared to the vehicle (Figure 2a). We used flow cytometry to verify the effects of VG161 or RT alone and the combination therapy on PD‐L1 expression on the surface of BC cells. In line with these findings, the upregulation of PD‐L1 caused by combination therapy was mainly attributed to VG161 in EMT‐6 cells, predominantly to RT in 4T1 cells, and equally to both agents in MDA‐MB‐231 cells (Figure 2b,c). This reinforces the need for the concurrent expression of checkpoint inhibitors. Our previous study confirmed that VG161/mVG161‐infected BC cells expressed transgenes IL‐12, IL‐15/IL‐15RA, and PD‐L1 peptide blocker (PD‐L1B) [35]. We next validated whether RT promoted these transgene expressions in BC cells. Consistent with the enhancement of viral replication, RT also increased the transgene expression of VG161/mVG161 in MDA‐MB‐231, EMT‐6, and 4T1 cells, including PD‐L1B, IL‐12, and IL‐15/IL‐15RA (Figure 2d). To determine whether the increase in transgene expression induced by RT is solely attributable to its role in promoting viral replication, or whether it may also involve the activation of transgene promoters, we calculated the ratio of the transgene payload concentration measured in the cell culture supernatant to the corresponding intracellular viral copy number. The results showed that there was no significant difference in the ratio of these transgenic payloads to viral copy numbers between VG161/mVG161 monotherapy and combination therapy (Figure S1d), suggesting that the role of RT in increasing transgene expression may be solely attributable to its role in promoting viral replication.

Next, we evaluated the role of these immunomodulatory transgenes in T‐cell activation. We cocultured human T cells with supernatants from MDA‐MB‐231 cells treated with VG160, VG161, RT, or VG161 plus RT, harvested the supernatant from the coculture system after 3 days, and detected pro‐inflammatory cytokines released during T‐cell activation. Compared to the other treatment groups, the levels of multiple pro‐inflammatory cytokines, including IL‐2, IL‐4, IL‐6, IL‐17A, tumor necrosis factor alpha (TNF‐α), and interferon gamma (IFN‐γ), were markedly elevated in the VG161 plus RT group (Figure 2e,f). In addition, co‐culture experiments of mouse T cells with the supernatants from 4T1 cells treated with mVG161, RT, or mVG161 plus RT showed that mVG161 combined with RT significantly promoted the proliferation and activation of T cells and greatly increased the secretion of multiple pro‐inflammatory cytokines (IL‐4, IL‐6, IL‐17A, and TNF‐α) compared with the other groups (Figure 2g–i). These data demonstrate that RT promotes immunomodulatory transgene expression of VG161, thus effectively activating T cells in vitro.

### 2.4 Combination of mVG161 and RT Augments ICD and Activates the cGAS‐STING Pathway in Bc

**FIGURE 3:**
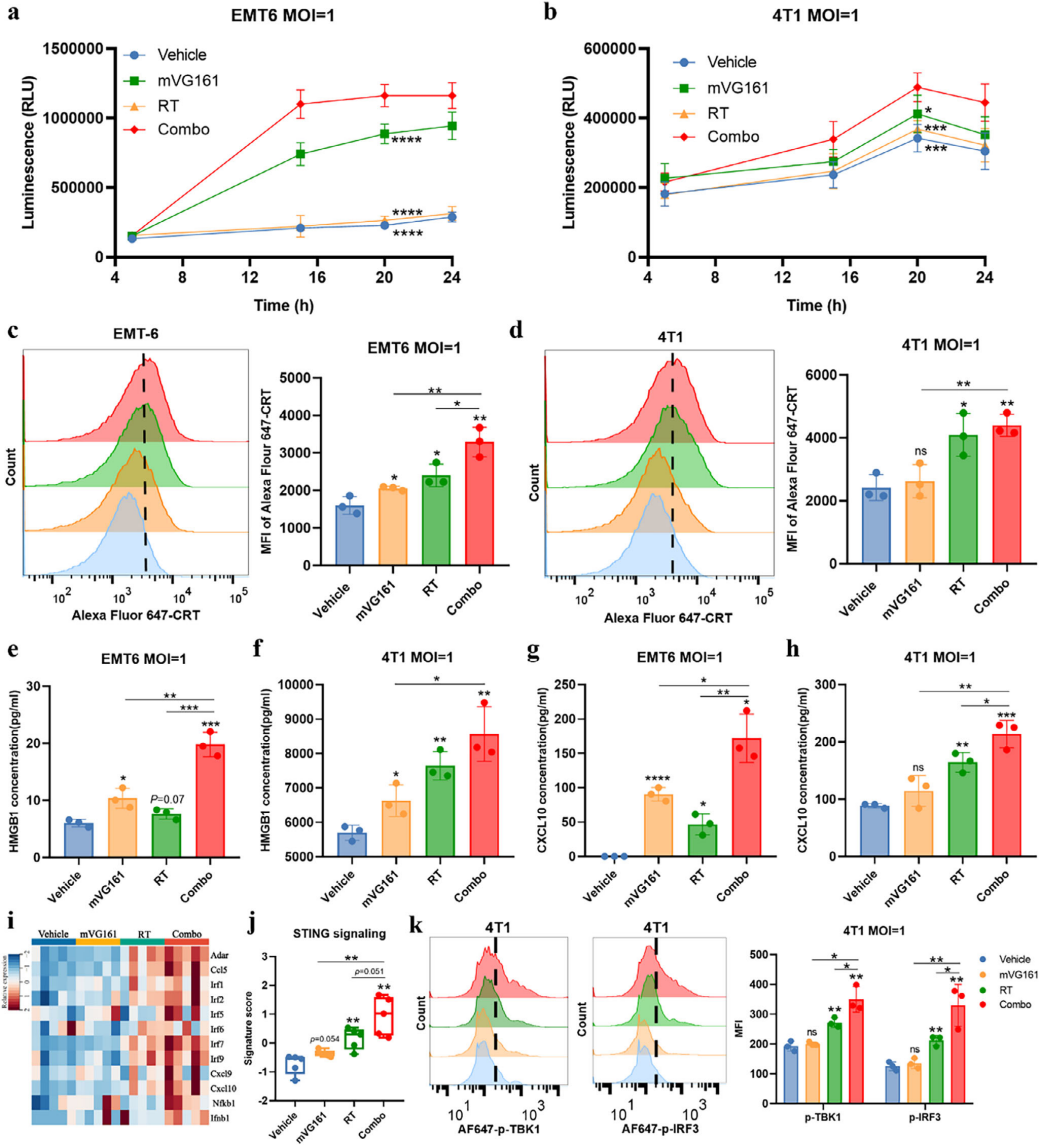
Combo therapy with mVG161 and RT augments immunogenic cell death and activates the cGAS‐STING pathway in BC. (a,b) ATP release from EMT‐6 and 4T1 tumor cells at the indicated time point after different treatments was detected (n = 6). (c,d) Representative images and quantification of the calreticulin (CRT) on the surface of EMT‐6 and 4T1 cells 24 h after various treatments was detected by flow cytometry, respectively (n = 3). (e,f) The HMGB1 level in the culture supernatants of EMT‐6 and 4T1 cells 48 h after different treatments was measured via ELISA (n = 3). (g,h) The CXCL10 level in the culture supernatants of EMT‐6 and 4T1 tumor cells 48 h after different treatments was measured by ELISA (n = 3). (i) Heatmap showing the expression levels of genes related to the STING signaling pathway in 4T1 tumor tissues 2 days after various treatments was detected by RNA‐seq (n = 5). (j) Quantification of gene signatures associated with STING signaling in 4T1 tumor tissues (n = 5). k) Representative images and quantification of the phosphorylated‐TBK1 (p‐TBK1) and phosphorylated‐IRF3 (p‐IRF3) levels in 4T1 cells 24 h after various treatments were detected by phosflow cytometry, respectively. The mean fluorescence intensity (MFI) of p‐TBK1 and p‐IRF3 was quantified (n = 3). All data are depicted as mean±SEM. The *p* values were calculated by Student's *t*‐test. Statistical significance compared to the vehicle group is indicated as follows: ^*^ *p* < 0.05, ^**^ *p* < 0.01, ^***^ *p* < 0.001, and ^****^ *p* < 0.0001. ns: not significant.

ICD induction is associated with the release of DAMPs that act through diverse mechanisms to increase antigen cross‐presentation and provide an inflammatory context for generating antigen‐specific T‐cell responses [37]. We next investigated whether the combination of mVG161 with RT‐induced cell death was associated with several common and widely accepted biochemical hallmarks of ICD, including the secretion of adenosine triphosphate (ATP), the exposure of calreticulin (CRT) on the cell surface, and the passive release of high mobility group box 1 (HMGB1). The results demonstrated that the combination of mVG161 and RT significantly enhanced ATP secretion from EMT‐6 and 4T1 cells, reaching a plateau or peak at 20 h (Figure 3a,b). Simultaneously, the combination of mVG161 with RT led to an enhancement in CRT surface exposure on EMT‐6 and 4T1 cells compared to the control and monotherapy groups (Figure 3c,d). In addition, a significant increase in the extracellular levels of HMGB1 was observed in both EMT‐6 and 4T1 cells following treatment with mVG161 in combination with RT (Figure 3e,f). These findings indicate that the combination of mVG161 and RT results in the release of DAMPs from tumor cells, suggestive of ICD.

To investigate the activation of the cGAS‐STING pathway by combination therapy, we analyzed the RNA‐seq data of 4T1 tumor tissues treated with either combination therapy or monotherapy. This analysis revealed that STING signaling‐related genes and signature scores were markedly upregulated in 4T1 tumor tissues receiving RT alone and were further increased following the combination of mVG161 with RT compared to the vehicle‐treated group (Figure 3i,j). Subsequently, we detected the phosphorylation levels of TBK‐1 and IRF‐3 in 4T1 cells using PhosFlow and observed that the levels of p‐TBK‐1 and p‐IRF‐3 were significantly increased in 4T1 cells subjected to RT, and further enhanced following the combination of mVG161 with RT (Figure 3k). Additionally, mVG161 plus RT markedly elevated CXCL10 secretion from EMT‐6 and 4T1 cells (Figure 3g,h). These findings demonstrate that combination therapy activates the cGAS‐STING signaling pathway in BC.

### 2.5 RT Enhances the Oncolytic Efficacy of mVG161 in Murine Breast Tumor Models

**FIGURE 4:**
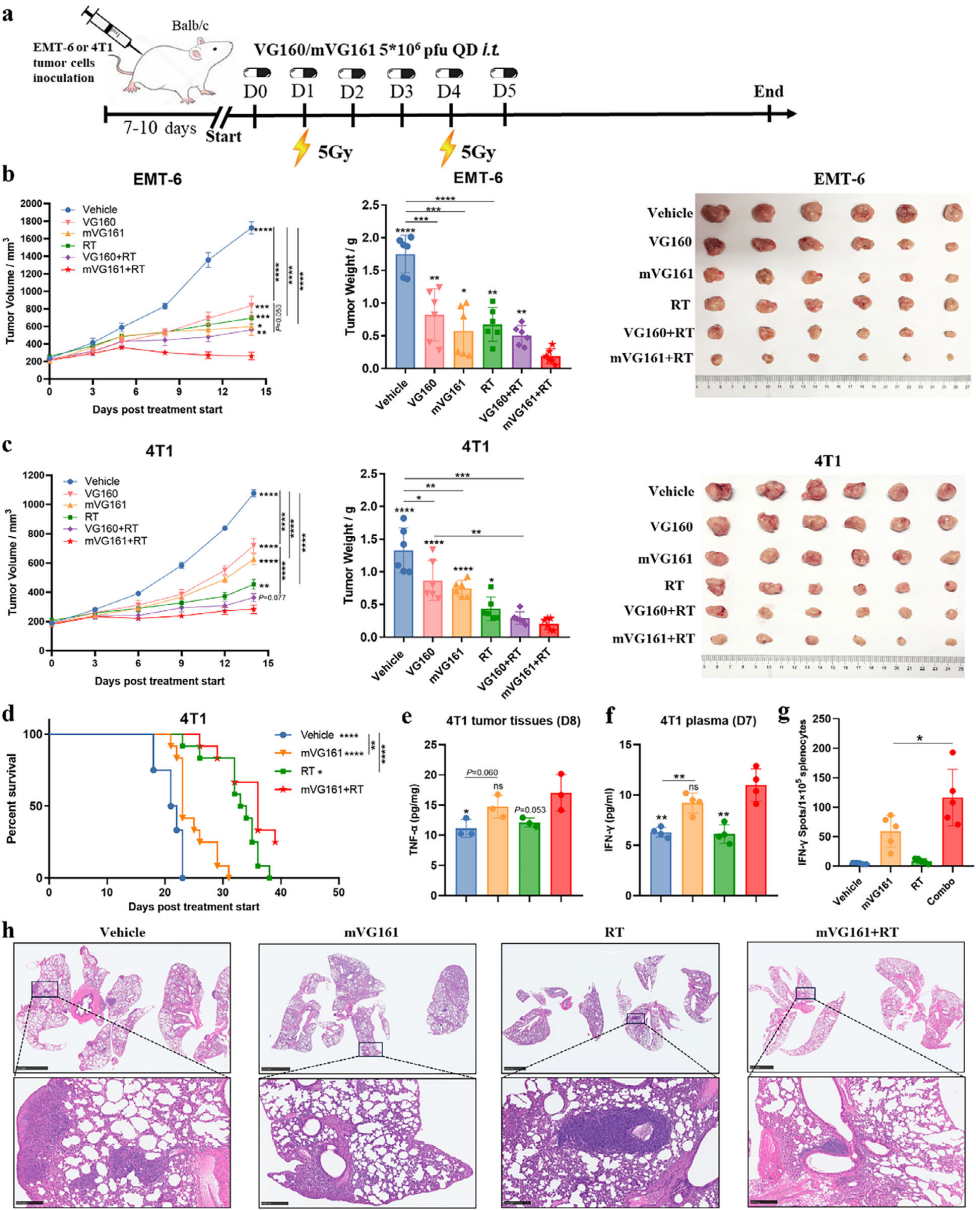
The synergistic antitumor efficacy between mVG161 and RT in murine BC models. (a) Schematic of treatment regimens for EMT‐6 and 4T1 orthotopic tumor model. (b,c) Tumor growth curves, tumor weights, and tumor photographs from EMT‐6 and 4T1 tumor‐bearing mice that received RT and/or mVG161/VG160 treatment, respectively (n = 6). (d) Kaplan–Meier survival curves for the orthotopic 4T1 tumor model by treatment with mVG161 combined with RT. Statistical significance compared to the RT plus Y332D group (n = 12). (e,f) The TNF‐α level in 4T1 tumor tissue homogenates and the IFN‐γ concentration in the plasma of BALB/c mice bearing 4T1 mammary carcinoma were measured by ELISA on day 8 or day 7 (n = 3 or 4). (g) IFN‐γ ELISpot to evaluate T cell activity in spleens from tumor‐bearing mice on day 8 following various treatments (n = 5). (h) The representative H&E staining images of lung tissues from mice bearing 4T1 orthotopic tumors that received combination or monotherapy treatments. The scale bar in the H&E staining images corresponds to 2.5 µm and 250 µm. All data are depicted as mean±SEM. Statistical analyses were conducted using Student's *t*‐test and *Log‐rank* test (d). Statistical significance compared to the mVG161 plus RT group is indicated as follows: ^*^ *p* < 0.05, ^**^ *p* < 0.01, ^***^ *p* < 0.001, and ^****^ *p* < 0.0001. ns: not significant.

To validate whether RT improves the oncolytic efficacy of mVG161 in vivo, EMT‐6 and 4T1 cells were inoculated into the right second mammary fat pad of BALB/c mice to establish orthotopic murine BC models. The treatment schedule is shown in Figure 4a. The EMT‐6 tumor model, characterized as a highly immunogenic and “immune‐inflamed” tumor type, typically is responsive to anti‐PD‐L1 therapy [38]. In this model, treatment with mVG161 or local RT significantly suppressed tumor growth and reduced tumor burden relative to the vehicle (Figure 4b). Notably, compared to the modified virus carrying immunostimulatory factors (mVG161), the oncolytic virus lacking immunostimulatory factors (VG160) exhibited slightly reduced antitumor efficacy (Figure 4b). Encouragingly, the combination of mVG161 and RT resulted in superior tumor growth inhibition and a greater reduction in tumor burden compared to the use of either mVG161 or RT alone in the EMT‐6 tumor model. Although no significant difference was observed compared with VG160 alone due to poor homogeneity in tumor size within the VG160 group, the combination of VG160 and RT showed a trend toward improved efficacy compared with VG160 alone. Importantly, mVG161 plus RT exhibited enhanced antitumor activity compared to VG160 plus RT in this tumor model (Figure 4b). The low‐immunogenicity 4T1 tumor model belongs to the “immune desert” subtype with poor tumor infiltration of immune cells and is relatively resistant to immune checkpoint inhibitors (ICIs) [39]. In this model, although there was no significant difference compared with VG160, the efficacy of the mVG161 showed a trend toward improvement compared with VG160 (Figure 4c). Consistent with the EMT‐6 model, mVG161 combined with RT demonstrated the strongest antitumor activity compared with other treatment groups in the 4T1 model (Figure 4c). Furthermore, the combination of VG160 and RT demonstrated significantly superior efficacy in the 4T1 model relative to VG160 alone. Additionally, mVG161 plus RT therapy significantly prolonged survival (median survival, 36 days) compared to mVG161 (23 days) or RT (33.5 days) (Figure 4d). Similar to the in vitro findings, in 4T1 tumor tissue, the combination of mVG161 and RT resulted in a 4.8‐fold increase in the expression of the viral gene qICP27 compared to mVG161 monotherapy without irradiation, implying that RT promotes the replication of mVG161 in tumor tissue (Figure S1e). Moreover, we found that combination therapy with mVG161 and RT significantly increased TNF‐α levels in 4T1 tumor tissues and IFN‐γ levels in plasma relative to the vehicle or RT and slightly elevated compared to mVG161 alone (Figure 4e,f). We performed IFN‐γ ELISpot assay on splenocytes collected from 4T1 tumor‐bearing mice 2 days after the final treatment and stimulated with 4T1 cells. The results suggested that the number of IFN‐γ‐secreting cells was significantly increased with mVG161 compared to the vehicle or RT alone and further enhanced after combination therapy with mVG161 and RT relative to mVG161 monotherapy (Figure 4g). These findings suggest that RT further amplifies the antitumor immunity elicited by mVG161. Additionally, we investigated whether combination therapy suppresses spontaneous metastasis in an orthotopic 4T1 BC model. HE staining showed that combination therapy with mVG161 and RT more effectively reduced the incidence of spontaneous lung tumor metastases than treatment with mVG161 or RT alone (Figure 4h).

### 2.6 The mVG161 Combined with RT Alters the Immunity‐Associated Genes Expression Profile

**FIGURE 5:**
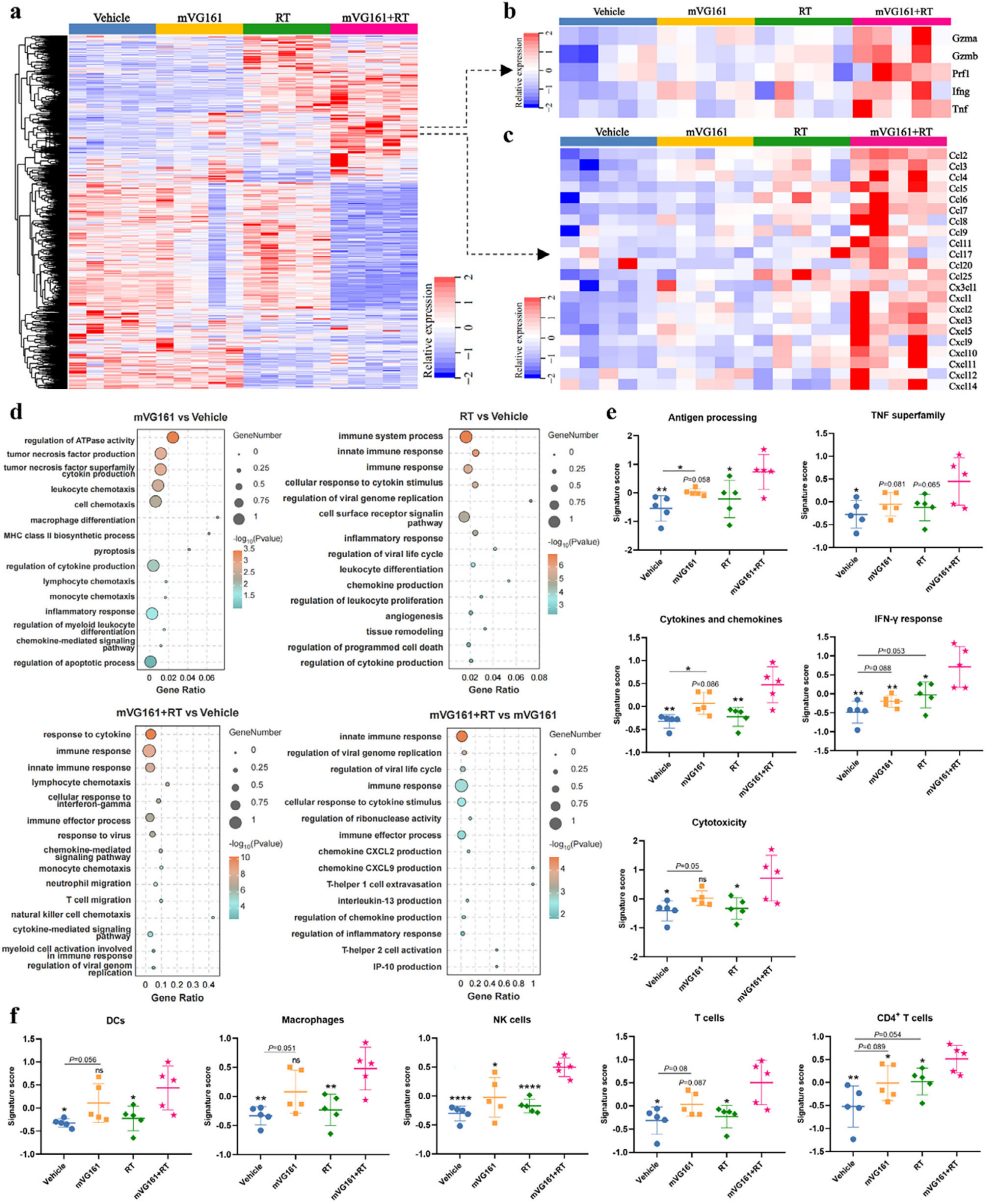
Therapy combining mVG161 with RT promotes the expression of immune signature genes as revealed by RNA‐seq. (a) Heatmap depicting the relative expression of differentially expressed genes (DEGs) among various treatment groups (n = 5). (b,c) Heatmap illustrating the relative expression of cytotoxicity‐related genes and proinflammatory chemokines among various treatment groups (n = 5). (d) GO biological process enrichment analysis based on DEGs between different treatment groups (mVG161 vs Vehicle, RT vs Vehicle, mVG161+RT vs Vehicle, mVG161+RT vs mVG161, respectively). (e,f) Quantification of signature genes associated with antigen processing, TNF superfamily, cytokines and chemokines, IFN‐γ response, cytotoxicity, DCs, macrophages, NK cells, T cells, and CD4^+^ T cells (n = 5). Quantitative data are mean±SEM. Statistical analyses were conducted using Student's *t*‐test. ^*^ *p* < 0.05, ^**^ *p* < 0.01, and ^****^ *p* < 0.0001 indicate significant differences compared to the mVG161 plus RT group. ns: not significant.

To comprehensively dissect the molecular mechanisms underlying the improved therapeutic efficacy observed with the combination of mVG161 and RT, we conducted RNA sequencing analysis of 4T1 tumor tissue samples. The differential expression gene (DEG) map revealed that RT contributed to most, but not all, of the gene expression changes observed in the mVG161 plus RT therapy group (Figure 5a). Notably, treatment with mVG161 and RT markedly altered the expression levels of a subset of genes that exhibited different expression patterns with RT or mVG161 alone, suggesting a synergistic mechanism at the gene level with the combination treatment (Figure 5a). Among these DEGs, genes encoding immune cytotoxicity‐related molecules such as GzmA (Granzyme A), GzmB (Granzyme B), Prf1 (Perforin), Ifng (IFN‐𝛾), and Tnf (tumor necrosis factor‐alpha) were upregulated in the combination therapy, which are critical mediators of CD8^+^ T cells and NK cells cytotoxicity and macrophage functions (Figure 5b). In addition, the combination of mVG161 and RT resulted in significantly higher expression of chemokine‐encoding genes, including Ccl2, Ccl5, Cxcl9, Cxcl10, and Cxcl11, which mediate monocyte and T cell intratumoral infiltration (Figure 5c). Gene Ontology (GO) biological process enrichment analysis based on the DEGs was performed to explore the effects of combination treatment on biological pathways. The results showed significant enrichment of pathways related to the immune response, innate immune response, inflammatory response, cytokine and chemokine production, cytokine/chemokine‐mediated signaling pathway, T cell and NK cell chemotaxis, and monocyte chemotaxis in the combination therapy (Figure 5d). In particular, some terms related to viral replication were significantly enriched in the combination therapy and RT groups, including regulation of viral genome replication and regulation of the viral life cycle (Figure 5d). Furthermore, we quantified the expression scores of signature genes associated with specific immunity biological functions and distinct immune cell types. Our data illustrated that mVG161 alone significantly increased the signature scores of antigen processing and cytokine and chemokine, and slightly enhanced the signature scores of TNF superfamily, IFN‐γ response, cytotoxicity, DCs, macrophages, NK cells, T cells, and CD4^+^ T cells compared to the vehicle (Figure 5e,f). Encouragingly, when combined with RT, mVG161 substantially upregulated all of these signature scores, which were the highest compared to the other treatment groups (Figure 5e,f).

### 2.7 Therapy Combining mVG161 with RT Increases Tumor‐Infiltrating Lymphocytes (TILs) and Inhibits Tumor Angiogenesis

**FIGURE 6:**
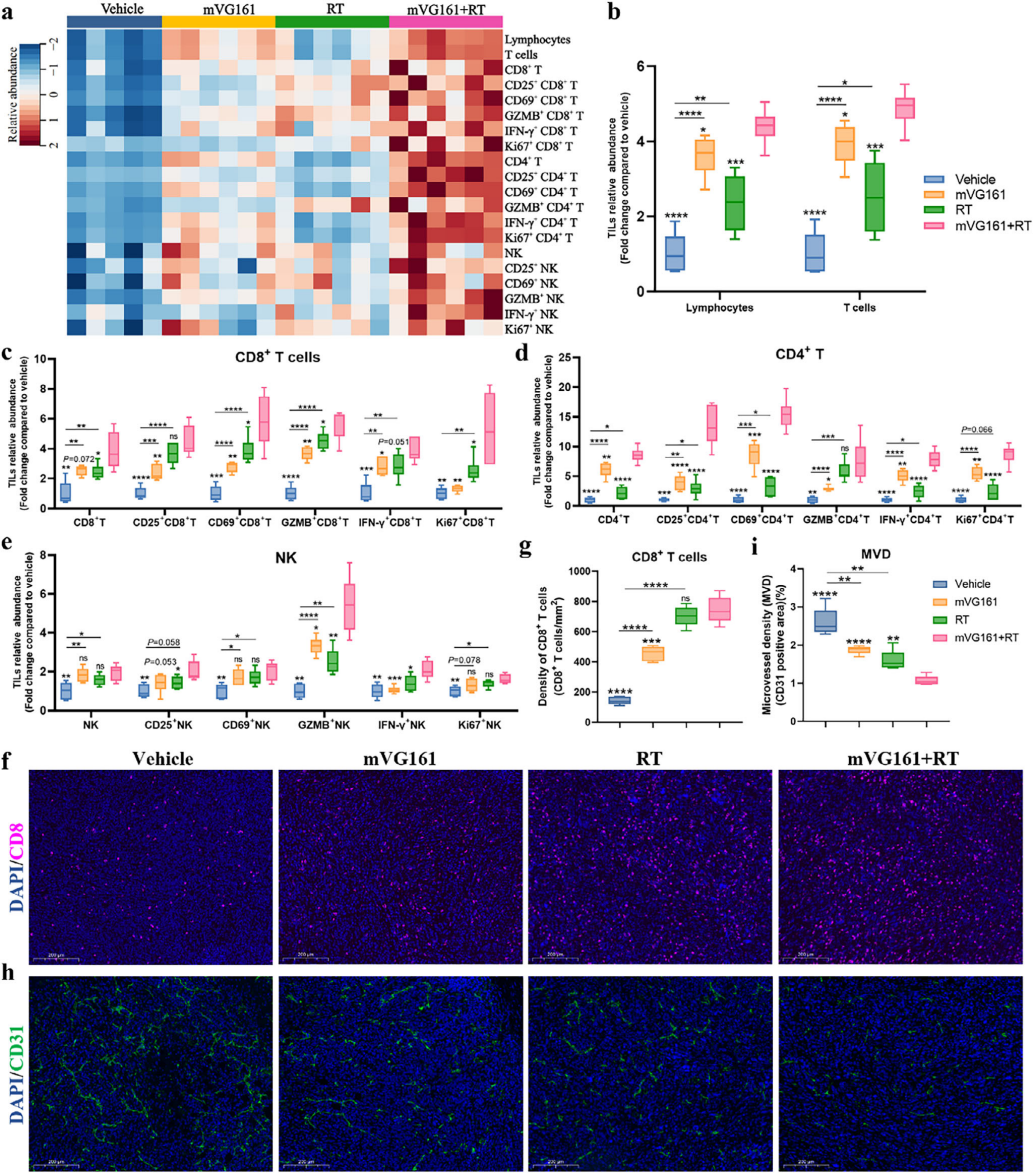
Combo therapy with mVG161 and RT enhances the abundances of TILs in the 4T1 tumor model. (a) Heatmap illustrating the relative abundances of TILs via flow cytometry (n = 5 or 6). (b–e) The relative abundances of TILs within the TME were assessed by flow cytometry across treatment groups (n = 5 or 6). (f,g) Representative images and quantification of CD8 immunofluorescence staining (n = 5). (h,i) Representative images and quantification of CD31 immunofluorescence staining (n = 5). The scale bar in the immunofluorescence images is 200 µm. Boxplots display minimum and maximum values with the median. Statistical analyses were conducted using Student's *t*‐test. ^*^ *p* < 0.05, ^**^ *p* < 0.01, ^***^ *p* < 0.001, and ^****^ *p* < 0.0001 indicate significant differences compared to the mVG161 plus RT group. ns: not significant.

To explore the impact of mVG161 combined with RT on tumor immune microenvironment (TIME) in the 4T1 tumor model with poor immune cell infiltration, we performed flow cytometry to characterize the abundance and activation state of TILs in 4T1 tumor tissues. Compared to monotherapy, combination therapy significantly increased the abundance of lymphocytes, CD3^+^ T, CD8^+^ T, activated CD8^+^ T (CD25^+^/CD69^+^ CD8^+^), cytotoxic CD8^+^ T (Gzmb^+^/IFN‐γ^+^ CD8^+^ T), proliferating CD8^+^ T (Ki67^+^ CD8^+^ T), CD4^+^ T, activated CD4^+^ T (CD25^+^/CD69^+^ CD4^+^ T), cytotoxic CD4^+^ T (Gzmb^+^/IFN‐γ^+^ CD4^+^ T), and proliferating CD4^+^ T (Ki67^+^ CD4^+^ T) cells (Figure 6a–d). Similarly, IF analysis of 4T1 tumor tissues revealed an obvious increase in the density of CD8^+^ T cells with mVG161 plus RT treatment compared to mVG161 alone (Figure 6f,g). These results suggest that, within the context of combination therapy, RT mainly contributes to an increase in the abundance and activation of CD8^+^ T‐cells, whereas mVG161 predominantly facilitates the proliferation and activation of CD4^+^ T‐cells. In addition, flow cytometry data showed that the combination therapy significantly increased the density of NK, activated NK (CD25^+^/CD69^+^ NK), cytotoxic NK (Gzmb^+^/IFN‐γ^+^ NK), and proliferating NK (Ki67^+^ NK) cells (Figure 6a,e). Tumor angiogenesis was also investigated in the 4T1 tumor model using IF. Intratumoral microvessel density (MVD), a surrogate measure of angiogenesis in tumor models, was evaluated by CD31 staining. Relative to the intratumoral MVD in the vehicle group, MVD was moderately reduced with mVG161 and gradually decreased after RT and combination therapy (Figure 6h,i).

### 2.8 Combination Therapy with mVG161 and RT Augments the Abscopal Effect on Spontaneous 4T1 Lung Metastatic Disease via Increasing Lymphocytes in Lungs

**FIGURE 7:**
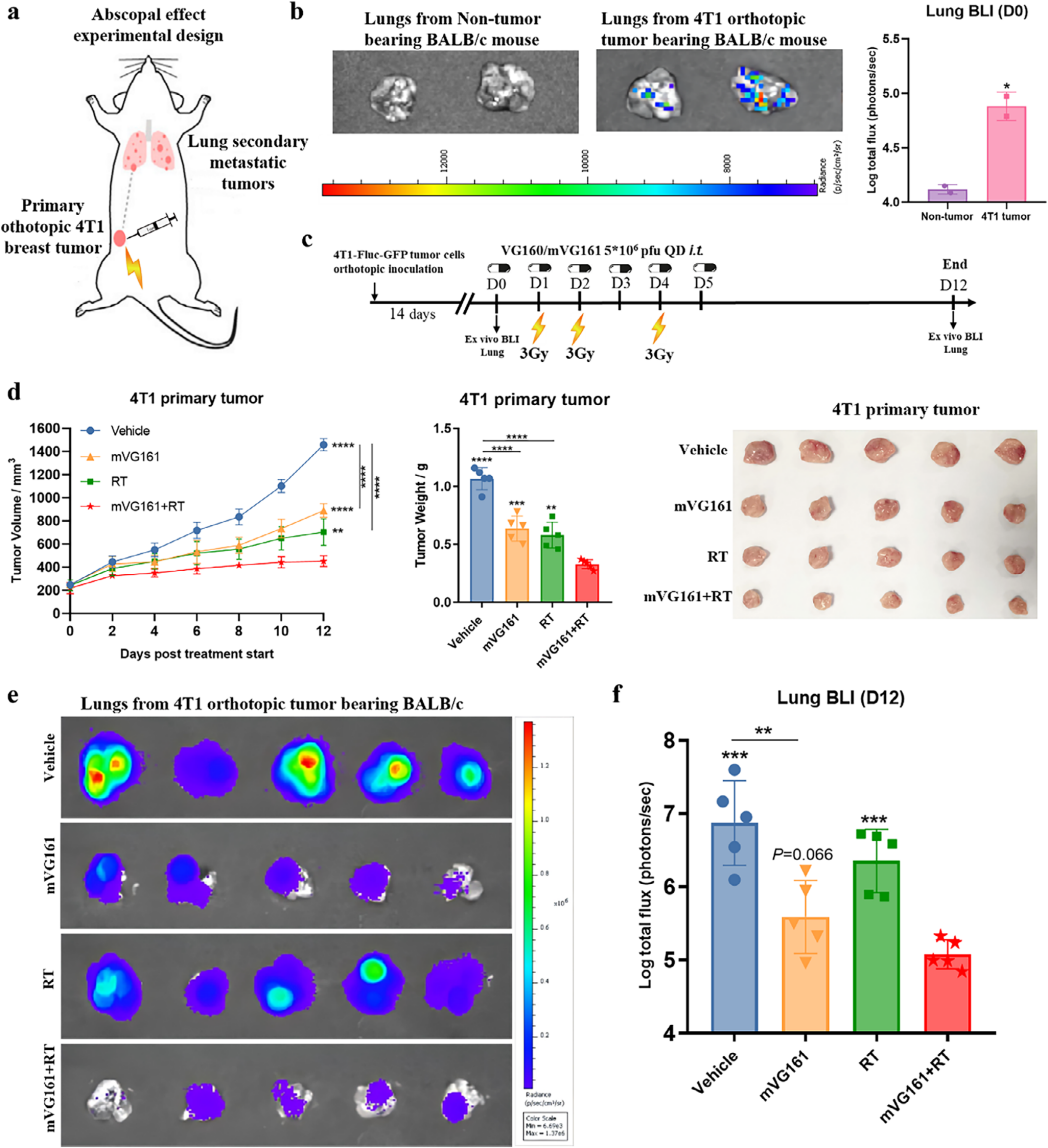
mVG161 plus RT therapy induces abscopal regression of spontaneous 4T1 lung metastases. (a) Schematic representation of the experimental design. (b) Ex vivo bioluminescence imaging (BLI) of lung metastases at day 0, with corresponding lung images and quantification data (n = 2). (c) Schematic diagram indicating the schedule and time points for the evaluation of secondary metastases and the administration of treatments. (d) The tumor growth curves, tumor weights, and photographs of orthotopic 4T1 primary tumors (n = 5). (e,f) Lung images and quantification of Ex vivo BLI of lung metastases on day 12 for each treatment group (n = 5). All data are depicted as mean±SEM. Statistical analyses were performed using Student's *t*‐test. Statistical significance compared to the mVG161 combined with RT group is denoted as follows: ^**^ *p* < 0.01, ^***^ *p* < 0.001, and ^****^ *p* < 0.0001. ns: not significant.

Local therapy can elicit antitumor effects on distant metastases, a phenomenon known as the abscopal effect. However, the abscopal effect induced by monotherapy is limited and rare. To investigate whether mVG161 combined with RT could amplify the abscopal effect, we used luciferase‐expressing 4T1 cells to establish a 4T1 spontaneous lung metastasis model (Figure 7a). The establishment of secondary disease was confirmed in the lungs of tumor‐bearing mice 14 days after tumor inoculation (day 0) using ex vivo bioluminescence imaging (BLI), whereas no luminescence was detected in the healthy lungs (Figure 7b). On the same day (day 0), the primary tumor started receiving mVG161 intratumoral injection (Figure 7a,c). Both mVG161 and RT monotherapies significantly inhibited primary orthotopic tumor growth compared to the vehicle, and mVG161 plus RT therapy exhibited superior efficacy in inhibiting primary tumor growth and reducing tumor burden relative to either monotherapy alone (Figure 7d). For secondary disease control, compared to the vehicle, mVG161 significantly reduced lung BLI on day 12, whereas RT did not yield a statistically significant effect (Figure 7e,f). Notably, combination therapy markedly diminished lung BLI on day 12 compared to RT alone and exhibited a tendency toward enhanced efficacy compared to mVG161 monotherapy (Figure 7e,f). These findings indicate that mVG161 and RT exhibit a synergistic effect in inhibiting the growth of primary tumors and the progression of secondary lung metastases.

**FIGURE 8:**
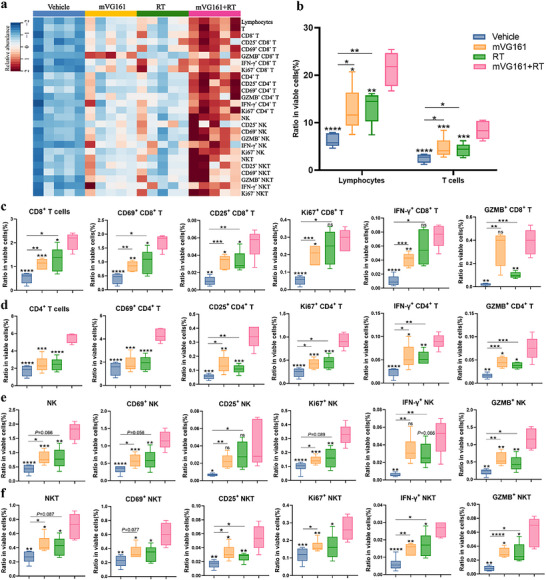
The combination of mVG161 and RT enhances lymphocyte infiltration in the lungs. (a) Heatmap of the relative abundance of lymphocytes in lung infiltrates as detected by flow cytometry (n = 5). (b–f) Quantification of various immune cell populations infiltrating lung tissues, as assessed by flow cytometry (n = 5). The proportion of infiltrating immune cells in the total live cells was calculated. Boxplots display minimum and maximum values with the median. Statistical analyses were conducted using Student's *t*‐test. ^*^ *p* < 0.05, ^**^ *p* < 0.01, ^***^ *p* < 0.001, and ^****^ *p* < 0.0001 indicate significant differences compared to the mVG161 plus RT group. ns: not significant.

The abscopal effect elicited by localized treatment is likely mediated by the activation of systemic antitumor immune responses. To investigate the mechanisms driving the abscopal effects observed following combined mVG161 and RT treatment, we conducted flow cytometric analysis of single‐cell suspensions derived from the whole lung tissues of mice bearing orthotopic 4T1 tumors. Compared to the vehicle and monotherapy, combination therapy significantly increased the percentage of infiltrating lymphocytes, CD3^+^ T, CD8^+^ T, activated CD8^+^ T (CD25^+^ CD8^+^ and CD69^+^ CD8^+^ T), cytotoxic CD8^+^ T (Gzmb^+^ CD8^+^ T and IFN‐γ^+^ CD8^+^ T), proliferating CD8^+^ T (Ki67^+^ CD8^+^ T), CD4^+^ T, activated CD4^+^ T (CD25^+^ CD4^+^ and CD69^+^ CD4^+^ T), cytotoxic CD4^+^ T (Gzmb^+^ CD4^+^ T and IFN‐γ^+^ CD4^+^ T), proliferating CD4^+^ T (Ki67^+^ CD4^+^ T), NK, activated NK (CD25^+^ NK and CD69^+^ NK), cytotoxic NK (IFN‐γ^+^ NK and Gzmb^+^ NK), proliferating NK (Ki67^+^ NK), NKT, activated NKT (CD25^+^ NKT and CD69^+^ NKT), cytotoxic NKT (IFN‐γ^+^ NK and Gzmb^+^ NK), and proliferating NKT (Ki67^+^ NKT) in total lung tissues (Figure 8a–f). These results indicate that T cells, NK cells, and NKT cells activated by combination therapy within the local TME migrate to the lungs via the bloodstream, thereby amplifying the abscopal effect.

### 2.9 The Depletion of CD8^+^ T Cells or NK Cells Impairs the Antitumor Efficacy of Combination Therapy with mVG161 and RT against Orthotopic 4T1 Tumors and Distant Lung Metastases

**FIGURE 9:**
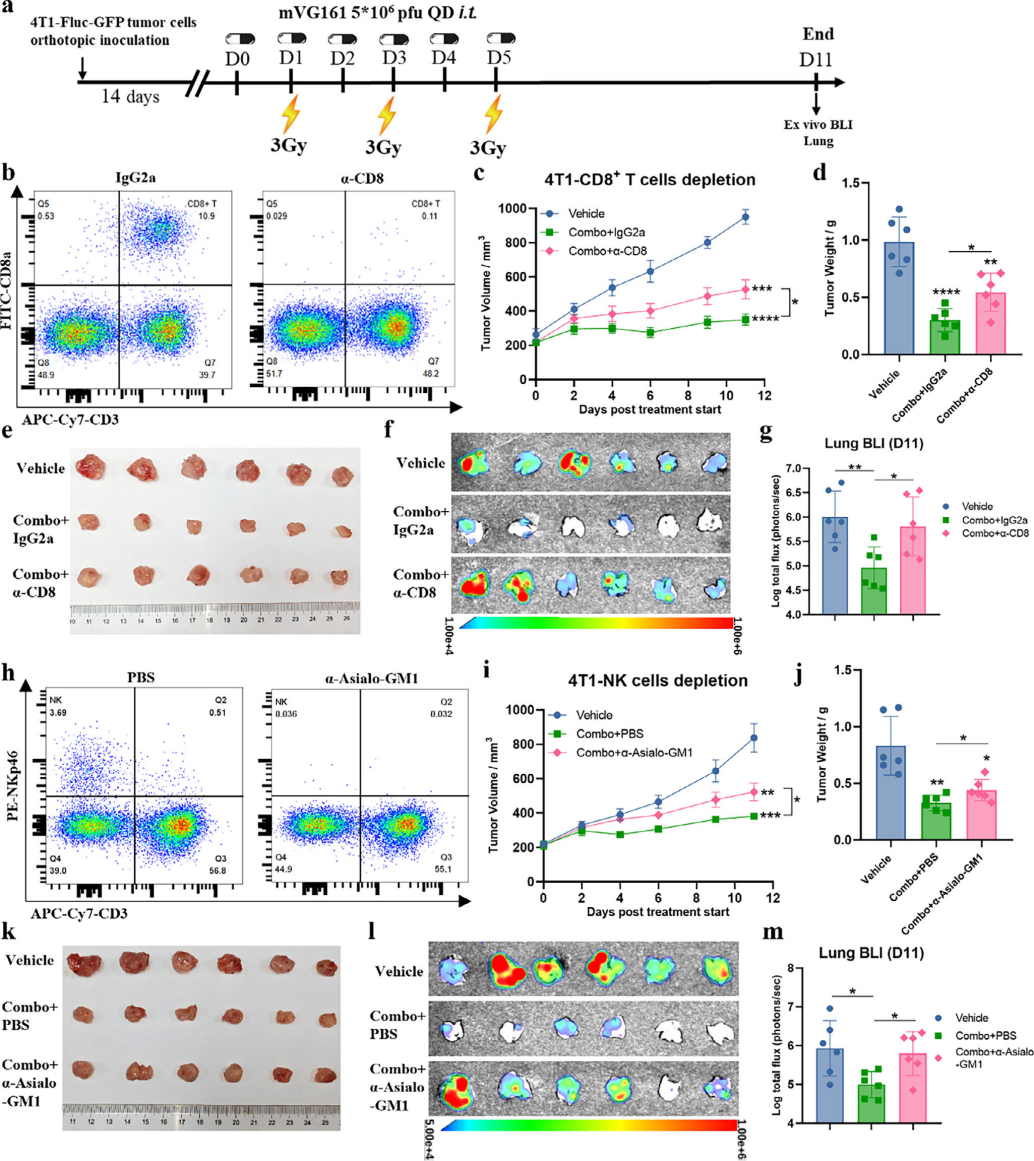
The depletion of CD8^+^ T cells or NK cells impairs the antitumor efficacy of combo therapy with mVG161 and RT against orthotopic 4T1 tumors and distant lung metastases. (a) Schematic diagram indicating the schedule and time points for the evaluation of lung metastases and the administration of treatments. (b,h) CD8^+^ T cells and NK cells depletion efficiency in peripheral blood was validated on day 3 after administration of the depletion reagent by flow cytometry, respectively. (c–e) Tumor growth curves, tumor weights, and photographs of orthotopic 4T1 tumors from the CD8^+^ T cells depletion experiment (n = 6). (f,g) Lung images and quantification of Ex vivo BLI of lung metastases on day 11 in the CD8^+^ T cells depletion experiment (n = 6). (i–k) Tumor growth curves, tumor weights, and photographs of orthotopic 4T1 tumors from the NK cells depletion experiment (n = 6). (l,m) Lung images and quantification of Ex vivo BLI of lung metastases on day 11 in the NK cells depletion experiment (n = 6). All data are mean±SEM. The *p* values were calculated by Student's *t*‐test. Significance is indicated as: ^*^ *p* < 0.05, ^**^ *p* < 0.01, ^***^ *p* < 0.001, and ^****^ *p* < 0.0001.

To further confirm the roles of CD8^+^ T cells and NK cells in the local antitumor effect and abscopal effects induced by combination therapy with mVG161 and RT, we specifically depleted CD8^+^ T cells and NK cells in the 4T1 tumor model using anti‐CD8a and anti‐Asialo‐GM1. The depletion efficiency of CD8^+^ T cells and NK cells was confirmed by flow cytometric analysis of peripheral blood (Figure 9a,h). In comparison to the non‐depleted control, depletion of CD8^+^ T cells markedly diminished the local antitumor efficacy and abscopal effect of the combination therapy in the 4T1 tumor model, leading to accelerated tumor growth and enhanced lung BLI (Figure 9c–g). Likewise, NK cell depletion resulted in faster tumor growth and stronger lung BLI than the non‐depleted control group, suggesting that NK cell depletion attenuated the local antitumor activity of the combination therapy and the abscopal effect it induced (Figure 9i–m). These findings indicate that both CD8^+^ T cells and NK cells play critical roles in mediating the local antitumor efficacy and abscopal effects elicited by combination therapy with mVG161 and RT in the 4T1 tumor model.

## 3 Discussion

Cytokine‐armed OVs have been actively pursued as a novel treatment strategy for cancer [40]. As the first OVs carrying multiple synergistic antitumor immune‐stimulating factors, local administration of VG161 not only has a direct oncolytic effect but also converts the immune “cold” phenotype into a “hot” one [10]. While wild‐type HSV is quite neurovirulent, the ICP34.5 deletion in VG161 that essentially eliminates neurovirulence also renders the specificity of the oncolytic HSV to tumor cells. Phosphorylation of the cellular translational initiation factor eIF‐2α can block the host's synthesis of proteins required for viral replication. The ICP34.5 protein can dephosphorylate eIF‐2α, thereby eliminating this inhibitory effect on protein synthesis and promoting viral replication. The ICP34.5 deletion, therefore, results in a reduction of viral replication. The mammalian homologue to the ICP34.5 is the GADD34 protein, which can functionally substitute for the ICP34.5 and is up‐regulated during DNA damage. RT is a well‐established modality in cancer treatment. In addition to its inherent tumoricidal activity, RT has been reported to enhance viral replication that results in increased tumor regression [17]. Interestingly, the HSV‐1 immediate‐early protein ICP0 inhibits post‐irradiation DNA damage repair, thereby enhancing tumor cell radiosensitivity [27, 28]. In a number of cancer types, HSV‐based OVs have a synergistic antitumor effect when combined with RT [29, 31, 32, 34, 41]. Moreover, a preclinical study has demonstrated that treatment with oncolytic HSV‐1, when followed within 6–9 h by administration of a clinically relevant fraction of radiation, resulted in the largest increase in viral replication and antitumor efficacy [24]. Given that viral replication depends on the temporal expression of viral genes and that the dose‐dependent cellular changes induced by RT, we first investigated the optimal timing and dose of RT in this combination regimen. The results revealed that a single administration of medium dose 5 Gy irradiation at 6 h following VG161 infection is the optimal regimen for combining oncolytic VG161 with RT, as this ensures that RT maximally increases VG161 replication in BC cells and tissues.

The underlying mechanism of increased viral replication in the presence of RT has been extensively studied. A widely‐accepted hypothesis is that RT increases the expression of the cellular DNA damage gene GADD34, resulting in greater viral replication and increased antitumor efficacy [23, 25]. Our results confirmed that GADD34 expression was increased in BC cells and 4T1 tumor tissues after irradiation exposure and that knocking down its expression before combination therapy significantly attenuated viral replication in BC cells. A region of the GADD34 protein shows significant structural homology to the ICP34.5 protein [42]. GADD34 also acts to prevent phosphorylation of eIF‐2α. Therefore, RT‐mediated upregulation of cellular GADD34 can functionally replace ICP34.5, resulting in increased viral protein synthesis and viral replication [31, 32, 33]. Another mechanism by which RT enhances replication of the ICP34.5‐deficient virus is that RT activates the late viral gene promoters via the p38 pathway, thereby enabling the synthesis of late viral proteins in sufficient amounts to augment viral replication [34]. Notably, our results revealed that RT also upregulated the HVEM expression on the surface of BC cells. HVEM is the receptor for HSV entry into tumor cells, and upregulation of HVEM by RT could enhance the efficiency of VG161 infection [43]. Further study revealed that HVEM knockdown before combination therapy significantly reduced viral DNA levels, indicating that the enhancement in VG161 induced by RT is partially dependent on the HVEM upregulation caused by RT. Although the upregulation of HVEM induced by RT is limited and HVEM is only one of the mediators of HSV‐1 entry into cells, our study provides new insights into the synergistic mechanisms underlying the interaction between HSV‐based OVs and RT, as no other studies have reported on the role of HVEM in combination therapy of RT and an HSV‐based OVs. Due to the diversity of genetic modifications among HSV‐based OVs, the mechanisms of interaction with RT vary. For instance, RT‐mediated upregulation of ribonucleotide reductase (RR) augmented the tumoricidal activity of G207 (ICP34.5^−^/ICP6^−^) but not of R3616 (ICP34.5^−^ only) [29]. ICP6 forms the large subunit of RR. Unlike the G207, T‐VEC has genetic deletions in ICP34.5 and ICP47, which otherwise blocks antigen presentation to MHC class I and II molecules by inhibiting TAP1 and TAP2 transporters [14, 26]. ICP47 deletion upregulates US11 and enable its expression earlier, which prevents PKR activation and eIF2α phosphorylation to maintain viral protein translation and simultaneously blocks IFN‐β signaling pathway to evade the host's antiviral response [44, 45, 46]. Therefore, the deletion of ICP47 complements the loss of ICP34.5 and enhances viral replication in tumor cells. Moreover, deletion of ICP47 promotes antigen presentation [14]. Although no direct evidence clarifies the synergy between ICP47‐deleted HSV‐1 and RT, we speculate that RT‐induced tumor antigens are more effectively presented, driving a systemic antitumor immune response. This aligns with a preclinical study showing that T‐VEC combined with RT suppresses both primary and distant tumors via robust systemic immunity [47]. Hence, it can be inferred that the effect of RT on HSV replication may depend on viral genotype (e.g., ICP34.5 and ICP47 status, US11 expression timing). Further studies are needed to assess whether RT modulates US11 expression and its role in ICP47‐deleted HSV‐1 plus RT. Besides, the impact of RT on other tumor‐restricted HSV strains with alternative attenuation mechanisms, such as replication‐conditional or re‐targeted OVs, remains unexplored.

A potential drawback to OV or RT treatment is the induction of counterregulatory checkpoint (PD‐L1) expression within the tumor [47, 48]. Our results demonstrated that combination therapy of RT and VG161 further increased PD‐L1 in BC cells compared to VG161 or RT alone. This potential inhibition of immune responses provides a strong mechanistic rationale for combining RT with OVs carrying a PD‐L1 blocker [47]. The PD‐L1 blocker, delivered by VG161, could combat PD‐L1 upregulation to some extent and is equivalent to an ICI, which can improve the immunosuppressive microenvironment of a tumor. Consistent with the enhancement of viral replication, our results indicated that the immunomodulatory transgenes of VG161 also increased by RT in BC cells, including PD‐L1B, IL‐15/IL‐15RA, and IL‐12, which upregulation is due to RT‐induced viral replication of VG161. They acted synergistically to promote antitumor immunity. Expression of the IL‐15/IL‐15RA complex could trigger NK cells and CD8^+^ T cells expansion and promote activation of antigen‐presenting cells (APCs) [49, 50, 51]. IL‐12 promotes polarization of antigen‐exposed T cells toward a pro‐inflammatory and antitumor T helper 1 (Th1) phenotype [52]. Multiple reports have indicated that systemic or local administration of IL‐12 leads to enhanced immune response and antitumor efficacy [53, 54, 55]. In this study, we confirmed the enhancement of these transgenic payloads of combination therapy on the proliferation and activation of T cells.

In parallel, RT or OVs alone were reported to induce ICD, resulting in the exposure or release of DAMPs, which include surface‐exposed CRT, secreted ATP, and released HMGB1, to ignite immune cells via being recognized by pattern recognition receptors on APCs [37, 56, 57, 58]. We verified that VG161 combined with RT treatment significantly elevated ATP secretion, CRT exposure, and HMGB1 release compared to VG161 or RT alone. Extracellular ATP secreted by dying cells operates as a prominent “find‐me” signal for DC precursors and macrophages upon binding to purinergic receptor P2Y2 and P2×7, thus facilitating the recruitment of myeloid cells to sites of active ICD [59]. Surface‐exposed CRT on the malignant cells undergoing ICD serves as an “eat‐me” signal that interacts with CD91 on APCs, including DCs, and facilitates the uptake of cell corpses and debris by APCs, thus providing them with an abundant source of antigenic material [60, 61]. Extracellular HMGB1 mediates robust adjuvant‐like effects by binding to TLR4 on DCs to stimulate efficient processing and cross‐presentation of tumor antigens from dying cells [62, 63]. Besides, RT has also been reported to activate the cGAS‐STING pathway in tumors [64]. We demonstrated that the combination therapy markedly promoted the expression of STING signaling‐related genes in 4T1 tumor tissues and the phosphorylation of TBK‐1 and IRF‐3 in 4T1 cells compared to mVG161 alone, suggesting the activation of the cGAS‐STING pathway by the combination therapy in BC. Notably, this activation was primarily driven by RT rather than mVG161. Besides, we also observed that VG161 combined with RT treatment obviously promoted the secretion of CXCL10, which can attract T cells and NK cells to infiltrate the tumor [65]. The cGAS‐STING pathway has long been regarded as the principal cellular antiviral host defense system. The antiviral machinery might be defective in tumor cells, which allows prolonged viral persistence and replication compared with infected normal cells [66, 67]. Theoretically, the increased activation of the STING pathway is expected also to increase antiviral responses. In fact, the cGAS‐STING pathway activated by RT is a double‐edged sword. It not only exerts a classic antiviral effect to limit the replication of OVs within tumor cells, but also shapes an inflammatory TME by promoting DCs maturation and T‐cell infiltration, thereby synergizing with OVs to launch an immune attack against tumor cells [64, 68]. Furthermore, the sequential therapy employed in this study, where the VG161 replicates fully within tumor cells first and then RT is administered, mitigated the impact of RT‐induced STING pathway activation on VG161 replication. Additionally, many HSV‐1 have evolved mechanisms to evade the host's antiviral defenses to facilitate their own replication. For example, the HSV‐1 protein US11 can inhibit the cGAS‐STING signaling pathway through various mechanisms, thereby evading the host's antiviral response [44, 45, 46]. Notably, our results revealed that although RT activated the STING pathway in 4T1 cells, RT has an overall enhancing effect on the replication of VG161 in 4T1 cells. Therefore, we infer that the activation of STING pathway by RT has a limited impact on viral replication, and is insufficient to affect the overall anti‐tumor efficacy of the combination therapy with VG161 and RT.

Based on the above synergistic mechanisms and in vitro findings, we investigated the antitumor efficacy of the combination of mVG161 with RT in murine BC models. Although mVG161 effectively retarded tumor growth in immune‐inflamed EMT‐6 tumor models, its antitumor effect was limited in the immune‐desert 4T1 tumor model. The combination therapy with mVG161 and RT dramatically boosted the oncolytic efficacy and viral replication of mVG161, especially in tumors with poor TILs infiltration. What's more, mVG161 plus RT treatment exhibited superior antitumor activity than VG160 plus RT treatment, which highlights the importance of the immunomodulatory payloads in promoting antitumor immunity. The explorations of the TME showed that the combination treatment significantly increased the abundances and activation of tumor‐infiltrating CD8^+^ T cells, CD4^+^ T cells, and NK cells. Sustained abnormal angiogenesis is one of the hallmarks of most cancers. Some OVs have been reported to attack tumor‐associated endothelial cells, resulting in reduced angiogenesis [2]. Our data suggested that mVG161 or RT monotherapy reduced tumor angiogenesis, and mVG161 plus RT therapy further reduced tumor angiogenesis. Our results indicated that this combination strategy successfully potentiated VG161 replication and amplified antitumor immunity, antagonized PD‐L1 upregulation caused by RT and VG161, and converted immunological “cold” tumors into a “hot” state.

Heretofore, we have demonstrated that mVG161 combined with RT had a synergistic antitumor effect on the primary tumors and induced pro‐inflammatory changes in the TME. It is widely accepted that in situ vaccines in primary tumors are the key mechanism transforming local effects into abscopal response [19]. The activated CTLs, Th1, and NKs in primary tumors move to tumor sites via the blood circulation and control metastatic tumor growth [19, 69]. Our results demonstrated that localized tumors treated with mVG161 plus RT significantly inhibited lung metastatic tumor growth compared to RT alone, indicating that combination therapy exhibits a synergistic effect not only in local antitumor effect but also in abscopal effects. Lung flow cytometry revealed that mVG161 combined with RT enhanced infiltration of CD3*^+^* T cells, CD4^+^ T cells, CD8^+^ T cells, NK cells, and NKT cells and promoted the activation, proliferation, and cytotoxicity of CD8^+^ T cells, CD4^+^ T cells, NK, and NKT cells in metastatic lungs. Higher numbers of tumor‐specific splenic T cells were also demonstrated by ELISpot assay in 4T1 tumor‐bearing mice treated with combination therapy compared to mVG161 or RT treated mice. The essential role of CD8^+^ T cells and NK cells in mediating the therapeutic efficacy elicited by combination therapy with mVG161 and RT was conclusively demonstrated via depletion experiments, wherein the depletion of CD8^+^ T cells or NK cells impaired the local antitumor efficacy and abscopal effect of combination therapy. These data indicate the combination of VG161 with RT induces a potent systemic antitumor immunity in vivo, resulting in an enhanced local antitumor efficacy and abscopal effect against preestablished, spontaneous lung metastases.

However, our study has several limitations that warrant further investigation, particularly the need to validate these findings in humanized NSG mouse models to support their clinical translation. Additionally, this study on the mechanisms by which RT promotes VG161 replication is exploratory and requires further experiments to clarify the extent of the contributions of the upregulation of GADD34 and HVEM by RT to the increase in VG161 replication.

## 4 Conclusions

**FIGURE 10:**
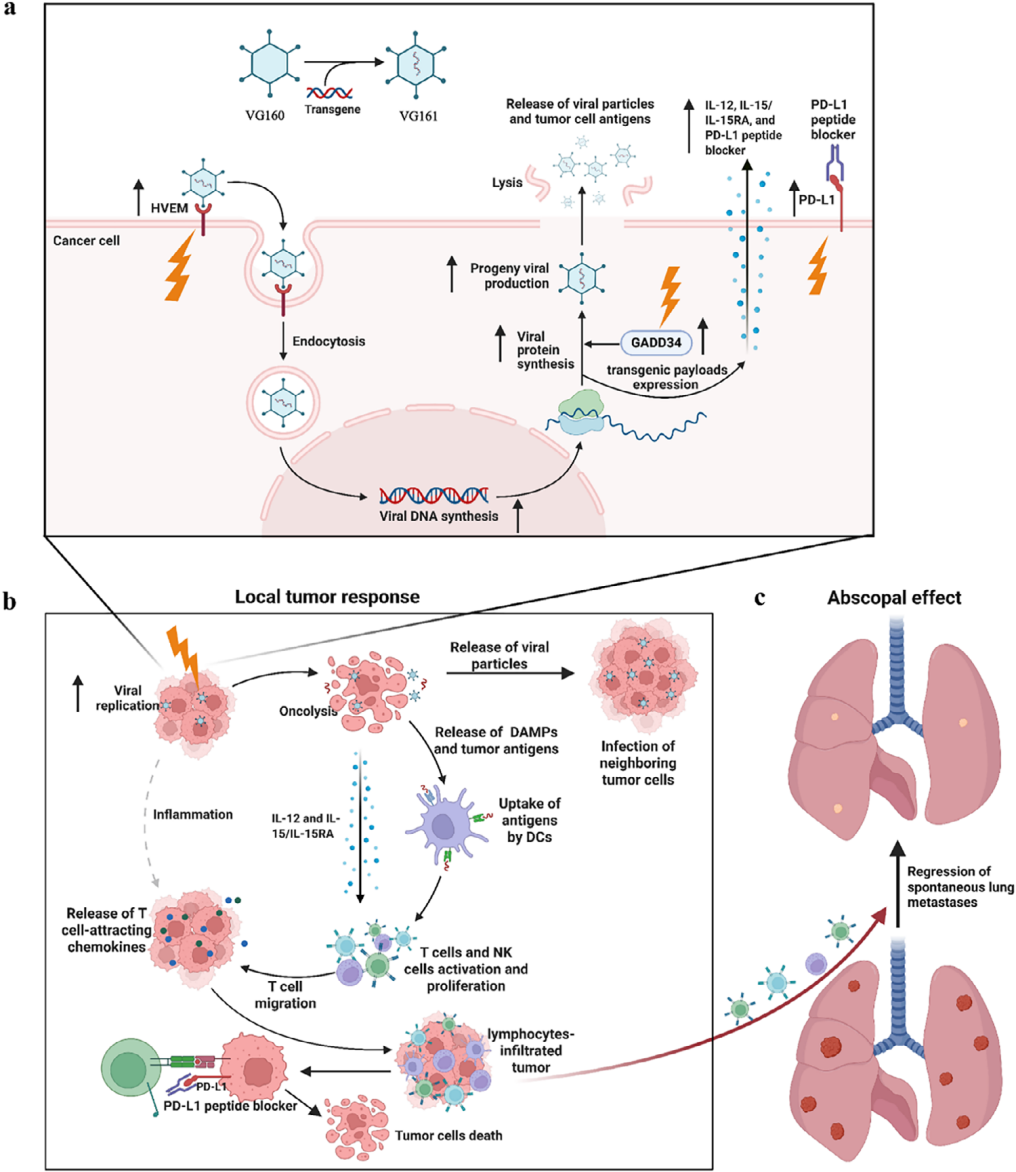
Schematic illustrating the synergistic mechanism of mVG161 combined with RT in the TME and abscopal metastatic niches. (a) RT potentiates the entry, replication and immune‐stimulating transgenes expression of ICP34.5‐deleted mVG161 in BC cells, which is mediated by upregulation of HVEM and GADD34 caused by RT. (b,c) The combination of mVG161 with RT acts synergistically to elicit a potent antitumor immunity and reprogram the TME toward the formation of an inflamed, or “hot” tumors, leading to the effective eradication of local tumors and an enhanced abscopal effect against preestablished, spontaneous lung metastatic lesions.

Our study suggests RT had a potentiating effect on viral replication and immunomodulatory transgene expression of ICP34.5‐deleted oncolytic herpesvirus VG161, which is mediated by upregulation of GADD34 and HVEM caused by RT (Figure 10a). In vivo, the combination therapy with VG161 and RT demonstrates superior antitumor effects compared with either monotherapy. Mechanistically, our findings reveal that this combination therapy elicits a potent antitumor immunity and reprograms the TME toward the formation of inflamed, or immune “hot” status, thereby effectively inhibiting local tumors and amplifying abscopal effect against preestablished, spontaneous lung metastases (Figure 10b,c). This abscopal effect is attributed to an enhanced influx and activation of cytotoxic CD8^+^ T cells, antitumor CD4^+^ T cells, NK cells, and NKT cells in metastatic lung niches. These findings establish a robust preclinical foundation and provide a compelling rationale for future clinical investigations of VG161 combined with RT, particularly in patients with non‐inflamed tumors.

## 5 Experimental Section

### 5.1 Cell Lines and Oncolytic Virus

Murine BC cell lines 4T1 and EMT‐6, derived from BALB/c mice, were cultured with RPMI‐1640 (Gibco) containing 10% fetal bovine serum (FBS) (Excell). Human BC cell line MDA‐MB‐231 was cultured with DMEM (Gibco) containing 10% FBS. These three cell lines tested negative for mycoplasma using MycoBlue Mycoplasma Detector (D101‐01, Vazyme) (Figure S1f). The constructions of VG160, VG161, and mVG161 were performed as described previously [10]. HSV‐1 strain 17 serves as the foundation for the generation of all recombinant viruses described herein, in which ICP34.5 was deleted. The oncolytic virus HSV‐1, designated VG161, was engineered to incorporate genes encoding human IL‐12, IL‐15, the IL‐15 receptor alpha (IL‐15RA) subunit isoform 1, as well as an expression cassette for a secretable PD‐L1 blocking peptide fused to the human IgG4 Fc (TF‐Fc). In contrast, VG160 lacks expression of human IL‐12, IL‐15, IL‐15RA, and the PD‐L1 blocking peptide. The murine analog, mVG161, is structurally identical to VG161 except for the replacement of human IL‐12 with its murine counterpart and the incorporation of a mouse‐specific PD‐L1 blocking peptide conjugated to mouse IgG1 Fc. The oncolytic viruses employed in this study were generously provided by Shanghai Virogin Biotech Co., Ltd.

### 5.2 Cell Viability Assay

MDA‐MB‐231, 4T1, and EMT‐6 cells (1 × 10^5^ cells/well) were seeded in 96‐well plates overnight at 37°C. The next day, the cells were infected with VG161 or mVG161 at MOI ranging from 0.5 to 8. After 48 h of infection, 10 µl CCK‐8 reagent (LK04, Dojindo) was added to each well and incubated for 2 h at 37°C before the absorbance was read at 450 nm.

### 5.3 Real‐Time Quantitative PCR (qPCR) and Reverse Transcription qPCR (RT‐qPCR)

Total RNA was extracted using the Animal Tissue/Cell Total RNA Extraction Kit (DP451, TIANGEN) following the manufacturer's instructions and quantified using an ultrafine spectrophotometer (N60 touch, IMPLEN). The reverse transcription reaction was performed using dNTPs and M‐MLV RT reverse transcriptase (M1701, Promega) according to the manufacturer's protocol. Genomic DNA was isolated using the FastPure Cell/Tissue DNA Isolation Mini Kit (DC102‐01, Vazyme). Real‐time qPCR was conducted with the iQ SYBR Green Supermix (1708882, Bio‐Rad) and pre‐designed primers (Tsingke Biotech) on the Applied Biosystems 7500 PCR System (Applied Biosystems, USA) according to the manufacturer's protocol. Before experiments, primer efficiency tests and melt curve analyses were performed to ensure the specificity of amplified products from all primer pairs. GAPDH was employed as a housekeeping gene to normalize the cycle threshold (Ct) values for the individual genes analyzed. The relative expression levels of each gene were determined using the 2^−ΔΔCT^ [ΔΔCt = ΔCt (test)−ΔCt (control)] method. The primer sequences utilized in this study are listed in Table S1.

### 5.4 Western Blotting

Tumor cells were lysed using RIPA buffer (P0013B, Beyotime). The supernatant was collected after centrifugation at 14 000 rpm for 15 min at 4°C, and the total protein concentration was measured using Pierce BCA Protein Assay Kit (A65453, ThermoFisher Scientific). A 30 µg protein sample was separated using SDS‐PAGE gel (NP0321BOX, Life Tech) and transferred to a polyvinylidene fluoride membrane (ISEQ00010, Millipore). Then, the membranes were blocked with 5% BSA for 1 h and incubated with the following primary antibodies: anti‐GADD34 (1:1000, 10449‐1‐AP, Proteintech) and anti‐GAPDH (1:1000, 5174, CST) at 4°C overnight. On the following day, the membranes were incubated with the secondary antibody Goat‐anti‐rabbit‐IgG‐HRP (1:2000, 7074, CST) for 1 h. SuperSignal West Pico PLUS (34577, ThermoFisher Scientific) was used for visualization, and the G: BOX Chemi X system was used for signal detection.

### 5.5 siRNA Transfection

The construction of si‐HVEM (5′‐GUAUGUGCUGACUGCCUAACATT‐3′ and 5′‐UGUUAGGCAGUCAGCACAUACTT‐3′), si‐GADD34 (5′‐GCCUGGGAGUACUACUCUAGATT‐3′ and 5′‐UCUAGAGUAGUACUCCCAGGCTT‐3′), and negative control (NC) (5′‐UUCUCCGAACGUGUCACGUTT‐3′ and 5′‐ACGUGACACGUUCGGAGAATT‐3′) were performed by Hecegene (Wuhan, China). The siRNA sequences involved in this study are listed in Table S1. For cell transfection, 4T1 and EMT‐6 cells (5 × 10^4^ cells/well) were seeded into 24‐well plates overnight at 37°C. Upon reaching approximately 70% confluence the following day, the cells were transfected with 15 pmol of si‐HVEM, si‐GADD34, or an NC using Lipofectamine 3000 reagent (L3000015, ThermoFisher Scientific) according to the manufacturer's protocol. Transfection efficiency was detected 48 h post‐transfection by RT‐qPCR and flow cytometry (Figure S1b,c). Subsequently, 24 h after transfection, the cells were infected with mVG161 at the indicated MOI and irradiated with 5 Gy 6 h following mVG161 treatment. At 24 h post‐RT, cells were harvested, genomic DNA was extracted, and qPCR was performed to determine the relative levels of viral qICP27 DNA.

### 5.6 Enzyme‐Linked Immunosorbent Assay (ELISA)

The levels of IL‐12p70, IL‐15/IL‐15RA complex, or PD‐L1 blocker in VG161 or mVG161‐infected cell culture supernatants were quantified using the following kits: Human IL‐12p70 Flex Set (558283, BD), Mouse IL‐12p70 Precoated ELISA Kit (1211203, Dakewe), Human IL‐15/IL‐15R Alpha Complex ELISA Kit (SEKH‐0281, Solarbio), Human IgG4 ELISA kit (SEKH‐0208, Solarbio), and Mouse IgG1 ELISA kit (JL20473, JONLNBIO). The concentrations of murine CXCL10 and high mobility group box 1 (HMGB1) in cell culture supernatants were quantified using the Mouse CXCL10/IP‐10 ELISA Kit (abs520013, Absin) and the Mouse HMGB1 ELISA Kit (E‐EL‐M0676, Elabscience), respectively. The levels of IFN‐γ in mouse serum and TNF‐α in tumor tissue homogenates were measured using the Mouse IFN‐γ Precoated ELISA kit (1210002, Dakewe) and Mouse TNF‐α Precoated ELISA kit (1217202, Dakewe), respectively. All procedures were executed following the manufacturer's guidelines.

### 5.7 The Proliferation and Activation of T Cells In Vitro

To investigate the impact of VG161/mVG161 payloads on the proliferation and activation of T cells, we cultured human or mouse T cells with conditioned medium derived from MDA‐MB‐231 or 4T1 pretreated with VG161/mVG161 and RT. Human T cells were obtained from the isolation of PBMCs by EasySep Human T Cell Isolation Kit (17951, STEMCELL), while murine T cells were obtained from the isolation of splenocytes from Balb/c mice by EasySep Mouse T Cell Isolation Kit (19851, STEMCELL). Before culture, murine T cells were labeled with carboxyfluorescein diacetate succinimidyl ester (CFSE) (2.5 µM, 65085084, ThermoFisher Scientific) and subsequently resuspended in conditioned medium from MDA‐MB‐231 or 4T1 cells pretreated with VG161/mVG161 and RT, respectively. The cell suspensions were then seeded into 96‐well flat‐bottom plates precoated with anti‐CD3 (0.5 µg/ml, 100302, BioLegend) and anti‐CD28 (0.5 µg/ml, 102116, BioLegend) antibodies. After 3 days, the cells and supernatants were harvested to assess T cell proliferation, activation, and cytokine secretion. Proliferation of CD8^+^ T cells was quantified via CFSE dilution assay, while activation status was determined by measuring the percentage of CD69^+^ CD4^+^ T cells and CD69^+^ CD8^+^ T cells using flow cytometry. The cytokine levels were evaluated employing the Cytometric Bead Array (CBA) Human Th1/Th2/Th17 Cytokine Kit (560484, BD) and Mouse Th1/Th2/Th17 Cytokine Kit (560485, BD).

### 5.8 The Realtime‐Glo Extracellular ATP Assay

4T1 and EMT‐6 cells (1 × 10^4^ cells/well) were seeded into 96‐well plates and incubated overnight at 37°C. On the following day, the culture medium in the plates was discarded, and fresh medium premixed with mVG161 (MOI = 1) and RealTime‐Glo Extracellular ATP reagent (M1701, Promega) was added. After 6 h, the cells were exposed to irradiation at a dose of 5 Gy. At the indicated times following RT, extracellular ATP release from the tumor cells was quantified by measuring luminescence.

### 5.9 Phospho‐Specific Flow Cytometry (Phosflow)

For the detection of intracellular phosphoprotein in tumor cells, we employed the Phosflow assay. 4T1 cells were detached using trypsinization, followed by fixation with prewarmed Phosflow fix buffer I (557870, BD Biosciences) at 37°C for 10 min, centrifuged, and the supernatant was removed. The cells were then permeabilized with ice‐cold Phosflow perm/wash buffer I (557885, BD Biosciences) for 20 min on ice, washed twice, centrifuged, and the supernatant was removed. Cells were resuspended in the staining buffer for intracellular phosphoprotein staining. Anti‐mouse p‐TBK1 Rabbit mAb (5483, CST, 1:500) or anti‐p‐IRF‐3 Rabbit mAb (29047, CST, 1:500) was added to each tube and incubated at room temperature for 1 h. After centrifugation in 1X PBS to wash and discard the supernatant, cells were resuspended in diluted fluorochrome‐conjugated secondary antibody (405307, BioLegend, 1:500) and incubated for 30 min at room temperature in the dark. Samples were finally resuspended in 100 µL staining buffer and analyzed using a BD FACSCelesta flow cytometer. FACS data were processed with FlowJo_V10 software.

### 5.10 Murine Tumor Models and Treatment

For the efficacy study, 5 × 10^5^ 4T1 and EMT‐6 cells were orthotopically implanted into the right second mammary fat pad of female BALB/c mice (6–8 weeks old). Upon tumor volume (TV) reaching approximately 200 mm^3^, mice were randomized into six treatment groups (n = 6 per group) based on TV and body weight, and treatment was initiated (day 0). The orthotopic 4T1 and EMT‐6 tumors received intratumoral (*i.t*.) injections of either vehicle (PBS) or VG160/mVG161(5 × 10^6^ PFU/mouse/day) for six consecutive days and were irradiated with two fractions of 5 Gy on day 1 and day 4 using the Small Animal Radiation Research Platform (SARRP, PXI X‐RAD 225Cx, USA) with an X‐ray energy of 225 kV, a current beam of 13 mA, a dose rate of 1.3 Gy/min and a source‐surface distance (SSD) of 60 cm. TV was measured every other day or every two days and calculated using the formula: TV = (length  ×  width [2])/2. Mice were euthanized when TV exceeded 2000 mm^3^ or at the study endpoint. For the abscopal effect study, 4T1‐Fluc‐GFP (1 × 10^5^) cells were orthotopically inoculated into the right fourth mammary fat pad of female BALB/c mice (6–7 weeks old) on day ‐14. On day 0, lungs from both 4T1 tumor‐bearing mice and non‐tumor‐bearing mice were harvested and placed in D‐fluorescein solution (300 mg/ml in saline) for ex vivo imaging to confirm the presence of pulmonary metastases. Subsequently, mice were randomized into four groups (n = 5 per group) according to the primary TV and body weight, and treatment was initiated. The primary tumors were injected intratumorally with either vehicle (PBS) or mVG161(5 × 10^6^ PFU/mouse/day) for six consecutive days and were irradiated with three fractions of 3 Gy on days 1, 2, and 4 using the SARRP under the same irradiation parameters described above. Mice were euthanized on day 12. All mice used in this experiment were 6–8‐week‐old female Balb/c mice.

### 5.11 ELISpot T Cell Activity Assay

Mouse IFN‐γ ELISpot assay (3321‐4APW‐10, Mabtech) was performed according to the manufacturer's instructions. Briefly, splenocytes isolated from treated mice were added to each well (1 × 10^5^ cells/well) and stimulated with 4T1 cells (5 × 10^3^ cells/well) and co‐cultured for 24 h to detect 4T1‐specific responses. Results were expressed as the number of spots per well.

### 5.12 In Vivo Depletion of CD8^+^ T Cells and NK Cells

To evaluate the dependence of the synergistic antitumor effect of mVG161 and RT on CD8^+^ T cells and NK cells, CD8^+^ T cells and NK cells were depleted in 4T1 tumor‐bearing mice using corresponding specific depletion antibodies. For the depletion of CD8^+^ T cells in female BALB/c mice (6–7 weeks old), 200 µg anti‐CD8a antibody (100763, Biolegend) was administered intraperitoneally (*i.p*.) for the initial injection on day ‐1 before treatment and then 100 µg every two days for subsequent injections. A rat IgG2a isotype control (400565, Biolegend) was used as a control. For NK cell depletion in female BALB/c mice (6–7 weeks old), 20 µg of polyclonal anti‐Asialo‐GM1 (146002, Biolegend) antibody was injected *i.p*. for the initial injection on day ‐1 before treatment and then 10 µg every 2 days for subsequent injections. PBS was administered as a control. The efficacy of depletion was confirmed by flow cytometry in peripheral blood.

### 5.13 Ex Vivo Bioluminescence Imaging (BLI)

The lungs were harvested and immersed in D‐luciferin solution (300 mg/ml in saline, ST196, Beyotime) for 2–3 min of imaging using an IVIS Spectrum (PerkinElmer) or SII Small Animal In Vivo Imaging System (AMI, Spectral Instruments Imaging). BLI images were analyzed using Living Image 4.3.1 (PerkinElmer) software or Aura In Vivo Imaging Software (Spectral Instruments Imaging), and total flux (photons/sec) was calculated and exported for all regions of interest (ROIs).

### 5.14 Flow Cytometry Assays

The expression of HVEM, CRT, and PD‐L1 on the surface of tumor cells was detected by flow cytometry. Adherent cells were dissociated into single‐cell suspensions using 0.05% trypsin and 0.02% EDTA and stained with fluorochrome‐conjugated anti‐HVEM (136303, BioLegend), anti‐calreticulin (62304, CST), and anti‐PD‐L1 (568085, BD) antibodies at 4°C for 30 min in the dark. After centrifugation in 1X PBS to wash and discard the supernatant, the cells were resuspended in 1X PBS and analyzed using a BD Accuri C6 or BD FACSCelesta flow cytometer. To detect immune cells infiltrating the tissue, 100 mg of 4T1 tumor tissues or whole lung tissues was mechanically disrupted, followed by enzymatically digested with collagenase B (0.5 mg/ml, 11088807001 Roche), DNase I (0.05 mg/ml, Sigma‐Aldrich), and hyaluronidase (0.5 mg/ml, H3506, Sigma‐Aldrich) at 37°C for 1 h, and finally filtered through 70 µm nylon cell filter to obtain single‐cell suspensions. The cell suspensions were treated with red blood cell lysis buffer (C3702, Beyotime) and washed. The cells were re‐suspended, supplemented with RPMI‐1640 medium with 10% FBS, and then added leukocyte activation cocktail with BD GolgiPlug (550583, BD), and then cultured in a 37°C CO_2_ incubator for 4 h. Following activation, the cells were harvested and washed with 1X PBS. Before staining, the cells were dyed with Fixable Viability Stain 780 (565388, BD Pharmingen) to distinguish viable cells and then blocked with Ultra‐LEAF Purified anti‐mouse CD16/32 (553141, BD). The cell suspensions were fluorescently stained with the following detection antibodies: anti‐CD45 (553079, BD), anti‐CD3ε (566494, BD), anti‐CD4 (563151, BD), anti‐CD8α (557959, BD), anti‐CD49b (740895, BD), anti‐Ki67 (563462, BD), anti‐CD69 (552879, BD), anti‐CD25 (557192, BD), anti‐Granzyme‐B (130‐116‐486, Miltenyi Biotec), anti‐IFN‐γ (563854, BD). Antibody staining of cell suspensions for flow cytometry analysis was performed following the antibody manufacturer's recommendations. The auxiliary reagents used in this assay were BD Pharmingen Stain Buffer (554658, BD) and Transcription Factor Buffer Set (562574, BD). The gating strategy for flow cytometry is shown in Figure S2. Flow cytometry was performed using Beckman CytoFLEX LX, and FACS data were analyzed by FlowJo_V10.

### 5.15 IF Staining

For the HSV‐1 ICP22 staining, 4T1 cells (2 × 10^4^ cells/well) were seeded into 24‐well plates overnight at 37°C. The next day, cells were infected with mVG161 at the indicated MOI. After 6 h, the cells were irradiated with 5 Gy. At 24 h post‐RT, cells were fixed with 4% paraformaldehyde for 30 min, permeabilized with 1% Triton‐X 100 for 20 min, and incubated with 5% BSA for 1 h. Cells were then incubated with anti‐HSV‐1 ICP22 (1:500, abs123385, absin) Rabbit mAb at 4°C overnight. Fluorescent secondary antibody (SA00003‐2, Proteintech, 1:250) was incubated at room temperature for 1 h. Cell nuclei were counterstained with DAPI (C2035S‐6, Beyotime). For the CD8 and CD31 staining, freshly isolated 4T1 tumor tissues were fixed in a 10% formalin solution for 24 h. The fixed tissues were then dehydrated, paraffin‐embedded, sectioned, and transferred to slides. IF staining based on tyramine signal amplification was performed according to the manufacturer's recommendations. Anti‐CD31 (ab28364, Abcam) and anti‐CD8 (98941, CST) were used in the IF assay. Images of IF were captured by fluorescence microscopy, and previewed via SlideViewer software, and ROIs were defined by two experienced pathologists. The quantitative analysis of IF images was conducted with ImageJ software.

### 5.16 Bulk RNA‐seq Assay

4T1 tumor tissues were harvested on day 7. Total RNA extraction using the Total RNA extraction Kit (DP419, TIANGEN). The quality and quantity of the purified RNA were determined by measuring the absorbance at 260 nm/280nm(A260/A280) using an Ultrafine spectrophotometer (N50 touch, IMPLEN). The integrity of the RNA was further verified by 1.0% agarose gel electrophoresis. RNA‐seq was conducted on the Illumina Novaseq 6000 platform (performed by Wuhan RuiXing). The reference genome utilized was Mus_musculus GRCm39, version M27 (Ensembl 104). DEGs between different groups were screened with the DESeq2 package in R software, with criteria set for fold change (FC) ≥ 2 or ≤ 0.5 and false discovery rate (FDR) < 0.05. Gene signatures were based on the nCounter Mouse PanCancer Immune Profiling Panel and published gene lists [70, 71]. Signature scores were defined as the mean Z‐score among all genes in each gene signature.

### 5.17 Statistical Analyses

Statistical analyses were conducted by GraphPad Prism V.8 software. Student's *t*‐test with or without Welch's correction and Mann–Whitney test were adopted to compare two groups. Survival Kaplan–Meier survival curves were compared by the *log‐rank* test. Data were presented as mean ± SE of mean (SEM) or SD. All tests were two‐sided, and *p* < 0.05 was regarded as significant.

## Author Contributions

**Lijuan Lyu** conducted the experiments, analyzed data, prepared the figures, and drafted the manuscript. **Ming Yi**, **Ji Chen**, and **Zeda Zhao** participated in experiments and revised the manuscript and figures. **Ruocen Liao**, **Yingnan Wang**, and **Shengtao Hu** participated in animal experiments and the data analysis. **Jun Ding**, **Ronghua Zhao**, **Yinan Shen**, and **William Jia** provided technical and material support and participated in the data interpretation. **Yongchang Wei**, **Kongming Wu**, and **Zhijun Dai** designed the work and supervised the study. All authors gave final approval of the version to be published and agreed to be accountable for all aspects of the work.

## Ethics Statement

The animal operations in this study were evaluated and approved by the Institutional Animal Care and Use Committee of The First Affiliated Hospital, College of Medicine, Zhejiang University (No. 202410344).

## Conflicts of Interest

J. D., RH. Z., and W. J. were employees of Shanghai Virogin Biotech Co., Ltd.

## Supporting information




**Supporting File**: advs76260‐sup‐0001‐SuppMat.doc.

## Data Availability

The data that support the findings of this study are available from the corresponding author upon reasonable request.
