# Supplementary material for: Radiotherapy Enhances the Oncolytic Efficacy of the Novel Oncolytic Herpesvirus VG161 and Amplifies Its Antitumor Immunity in Breast Cancer
Source: Adv Sci (Weinh). 2026 Jun 29:e23356. Online ahead of print. doi: 10.1002/advs.202523356 (PMC13337113; doi:10.1002/advs.202523356)
Supplement: Supplementary file 1 — Supporting File: advs76260‐sup‐0001‐SuppMat.doc. [file ADVS-9999-e23356-s001.doc]

**Supporting Information**

**Table S1. qPCR primer sequences used in this study.**

| Gene name | Forward | Reverse |
| --- | --- | --- |
| Gapdh | 5′-CGACTTCAACAGCAACTCCCACTCTTCC-3′ | 5′-TGGGTGGTCCAGGGTTTCTTACTCCTT-3′ |
| Gadd34 | 5′-GGCGGCTCAGATTGTTCAAAGC-3′ | 5′-CCAGACAGCAAGGAAATGGACTG-3′ |
| qICP27 | 5′-CGCCAAGAAAATTTCATCGAG-3′ | 5′-ACATCTTGCACCACGCCAG-3′ |
| IL15RA1 | 5′-CTCTCCAAG CTC CAA CAATACA-3′ | 5′-GAGGACTCGTGGCTAGAGAT-3′ |

**Table S2. siRNA sequences used in this study.**

| siRNA name | Sense strand sequence | Antisense strand sequence |
| --- | --- | --- |
| NC | 5′-UUCUCCGAACGUGUCACGUTT-3′ | 5′-ACGUGACACGUUCGGAGAATT-3′ |
| FAM-NC | 5′-UUCUCCGAACGUGUCACGUTT-3′ | 5′-ACGUGACACGUUCGGAGAATT-3′ |
| HVEM-164 | 5′-CCAUGUGCAACCCAGGUUACCTT-3′ | 5′-GGUAACCUGGGUUGCACAUGGTT-3′ |
| HVEM-132 | 5′-GGAGGAGUUCCUUGUGGGAGATT-3′ | 5′-UCUCCCACAAGGAACUCCUCCTT-3′ |
| HVEM-481 | 5′-GUAUGUGCUGACUGCCUAACATT-3′ | 5′-UGUUAGGCAGUCAGCACAUACTT-3′ |
| GADD34-328 | 5′-GUUGAUGAGUACAAUGCAAAGTT-3′ | 5′-CUUUGCAUUGUACUCAUCAACTT-3′ |
| GADD34-706 | 5′-GCCUGGGAGUACUACUCUAGATT-3′ | 5′-UCUAGAGUAGUACUCCCAGGCTT-3′ |
| GADD34-1725 | 5′-GGAGCAGUUUGCACGAGAUCGTT-3′ | 5′-CGAUCUCGUGCAAACUGCUCCTT-3′ |

**Table S3.** **The clones and catalog numbers of the antibody involved in this study.**

| Antibodies | Clones | Catalog numbers | Source |
| --- | --- | --- | --- |
| FITC anti-Mouse CD45 | 30-F11 | 553079 | BD |
| BB700 anti-Mouse CD3e | 145-2C11 | 566494 | BD |
| PE anti-aGranzyme B | REA226 | 130-116-486 | BD |
| PE-Cy7 anti-Mouse CD69 | H1.2F3 | 552879 | BD |
| APC anti-Mouse CD25 | PC61 | 557192 | BD |
| Alexa Fluor 700 anti-CD8a | 53-6.7 | 557959 | BD |
| BV510 anti-Ki-67 | B56 | 563462 | BD |
| BV605 anti-Mouse CD4 | RM4-5 | 563151 | BD |
| BV650 anti-Mouse IFN-γ | XMG1.2 | 563854 | BD |
| BV786 anti-Mouse CD49b | HMα2 | 740895 | BD |
| Rat anti-Mouse CD16/CD32 | 2.4G2 | 553141 | BD |
| BV421 anti-CD69 | H1.2F3 | 562920 | BD |
| APC anti-CD4 | RM4-5 | 561091 | BD |
| PE anti-CD274 (PD-L1) | 10F.9G2 | 568085 | BD |
| PE anti-HVEM | HMHV-1B18 | 136303 | Biolegend |
| Anti-GADD34 | Polyclonal | 10449-1-AP | Proteintech |
| Anti-GAPDH | D16H11 | 5174 | CST |
| Anti-CD3 | 145-2C11 | 100302 | Biolegend |
| Anti-CD28 | 37.51 | 102116 | Biolegend |
| Anti-p-TBK1 | D52C2 | 5483 | CST |
| Anti-p-IRF3 | D6O1M | 29047 | CST |
| Anti-CD8a | 53-6.7 | 100763 | Biolegend |
| Rat IgG2a | RTK2758 | 400565 | Biolegend |
| Anti-Asialo-GM1 | Poly21460 | 146002 | Biolegend |
| Anti-calreticulin | D3E6 | 62304 | CST |
| Anti-HSV-1 ICP22 | Polyclonal | abs123385 | Absin |
| Anti-CD31 | Polyclonal | ab28364 | Abcam |
| Anti-CD8 | D4W2Z | 98941 | CST |


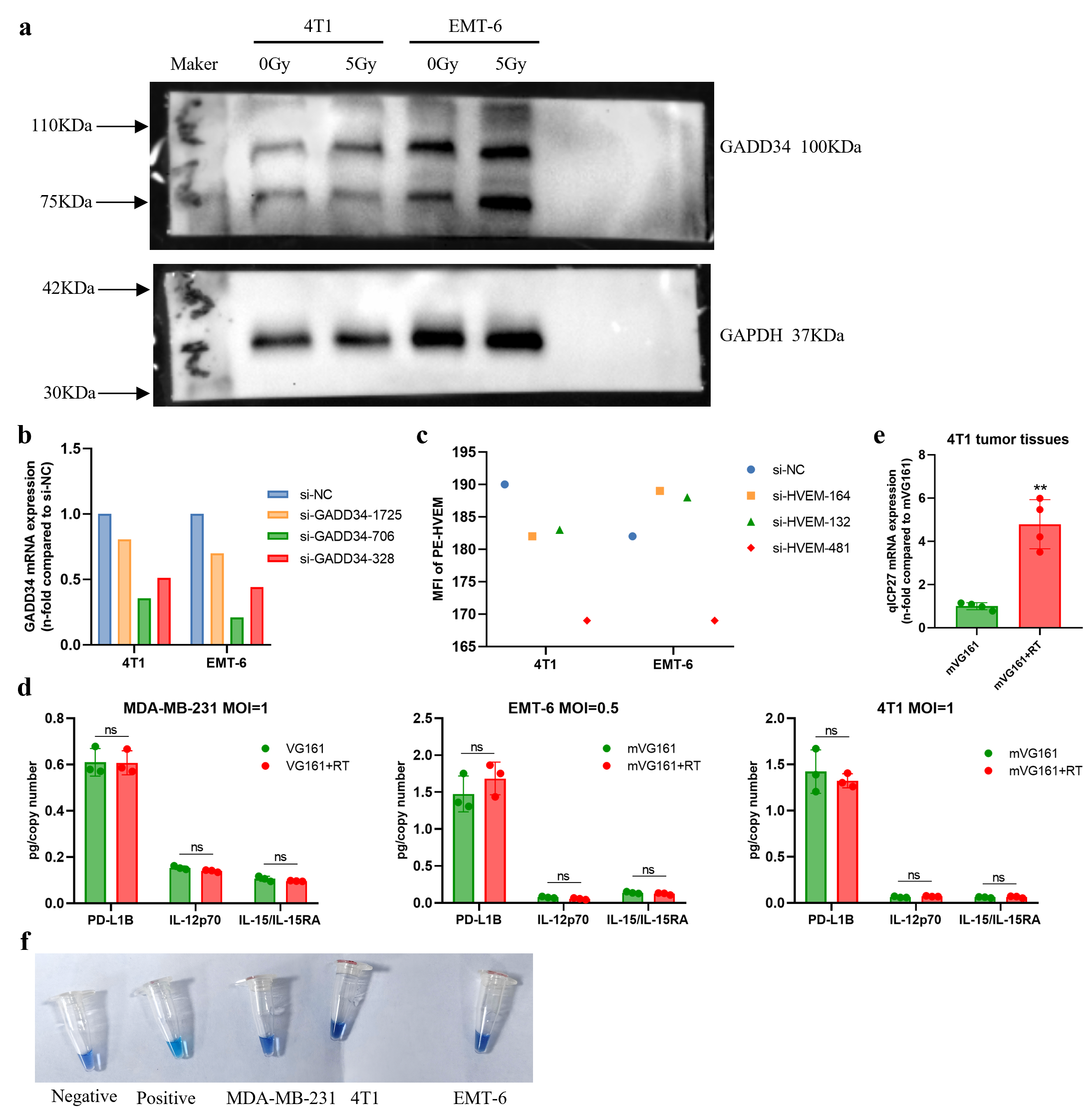


**Figure S1**. a) The full uncropped Gels and Blots images in Figure 1g. b) Detection of GADD34 expression in tumor cells via qRT-PCR 48 hours after siRNA transfection (n = 1). c) Detection of HVEM expression on the surface of tumor cells by flow cytometry 48 hours after siRNA transfection (n = 1). d) The ratio of transgenic cytokine levels in tumor cell culture supernatant to the DNA copy number of VG161 viral in tumor cells (n = 3). e) The HSV-1 qICP27 mRNA expressions in 4T1 tumor tissues 2 days after different treatments were confirmed by qRT-PCR (n = 3). f) The results of mycoplasma testing for the three BC cell lines involved in this study. Statistical analyses were conducted using Student’s *t*-test. Significance is indicated as: ***P* < 0.01; ns: not significant.


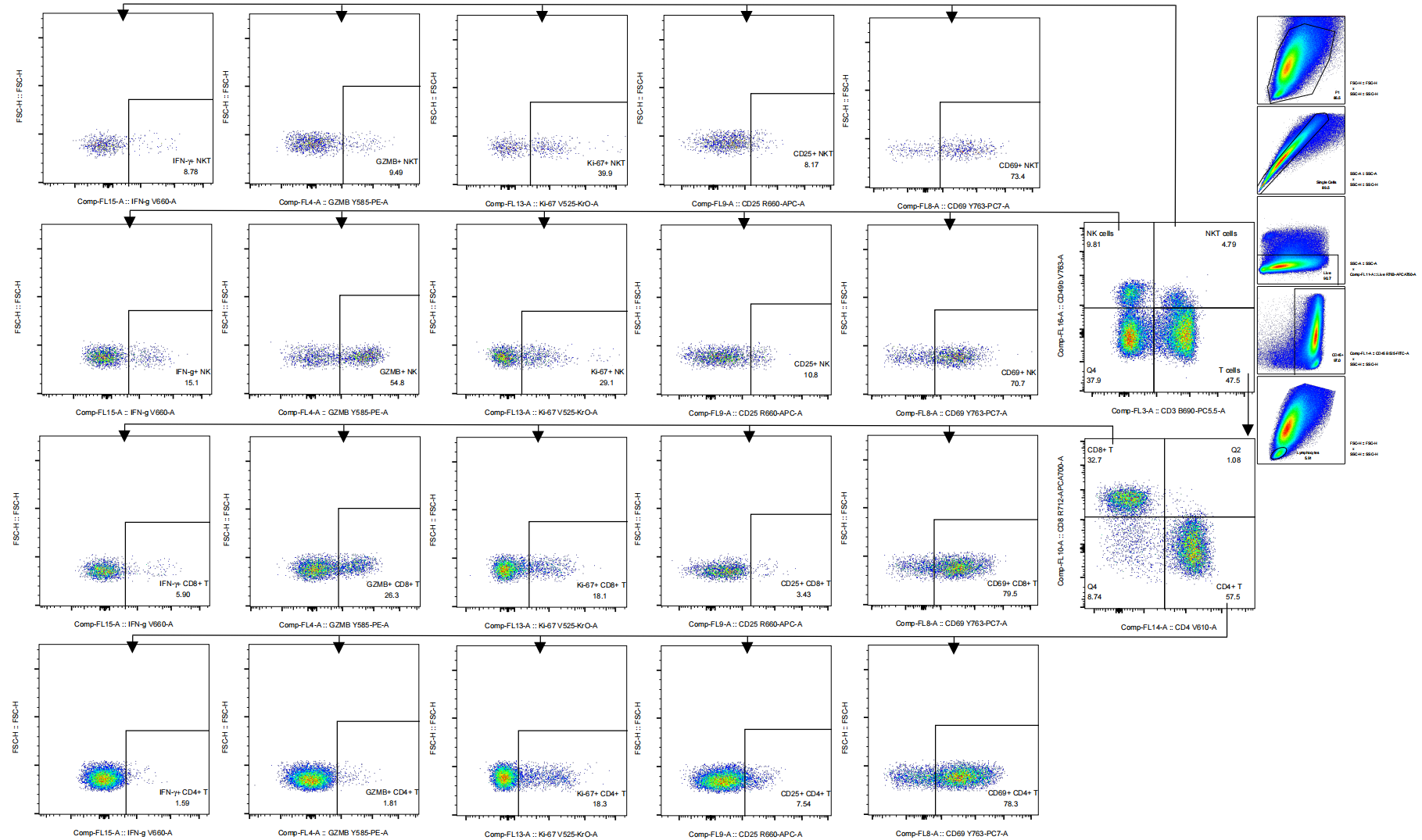


**Figure S2**. Flow cytometry gating strategies for identifying CD8^+^ T cells, CD4^+^ T cells, NK cells, and NKT cells.
